# Supplementary material for: Enteropathogenic Escherichia coli Stimulates Effector-Driven Rapid Caspase-4 Activation in Human Macrophages
Source: Cell Rep. 2019 Apr 23;27(4):1008–1017.e6. doi: 10.1016/j.celrep.2019.03.100 (PMC6486487; doi:10.1016/j.celrep.2019.03.100)
Supplement: Document S1. Figures S1–S5 and Table S1 [file mmc1.pdf]

Cell Reports, Volume *27*

Supplemental Information

Enteropathogenic *Escherichia coli* Stimulates Effector-Driven Rapid Caspase-4 Activation in Human Macrophages

Philippa J. Goddard, Julia Sanchez-Garrido, Sabrina L. Slater, Mohini Kalyan, David Ruano-Gallego, Olivier Marchès, Luis Ángel Fernández, Gad Frankel, and Avinash R. Shenoy

Goddard *et al* Figure S1

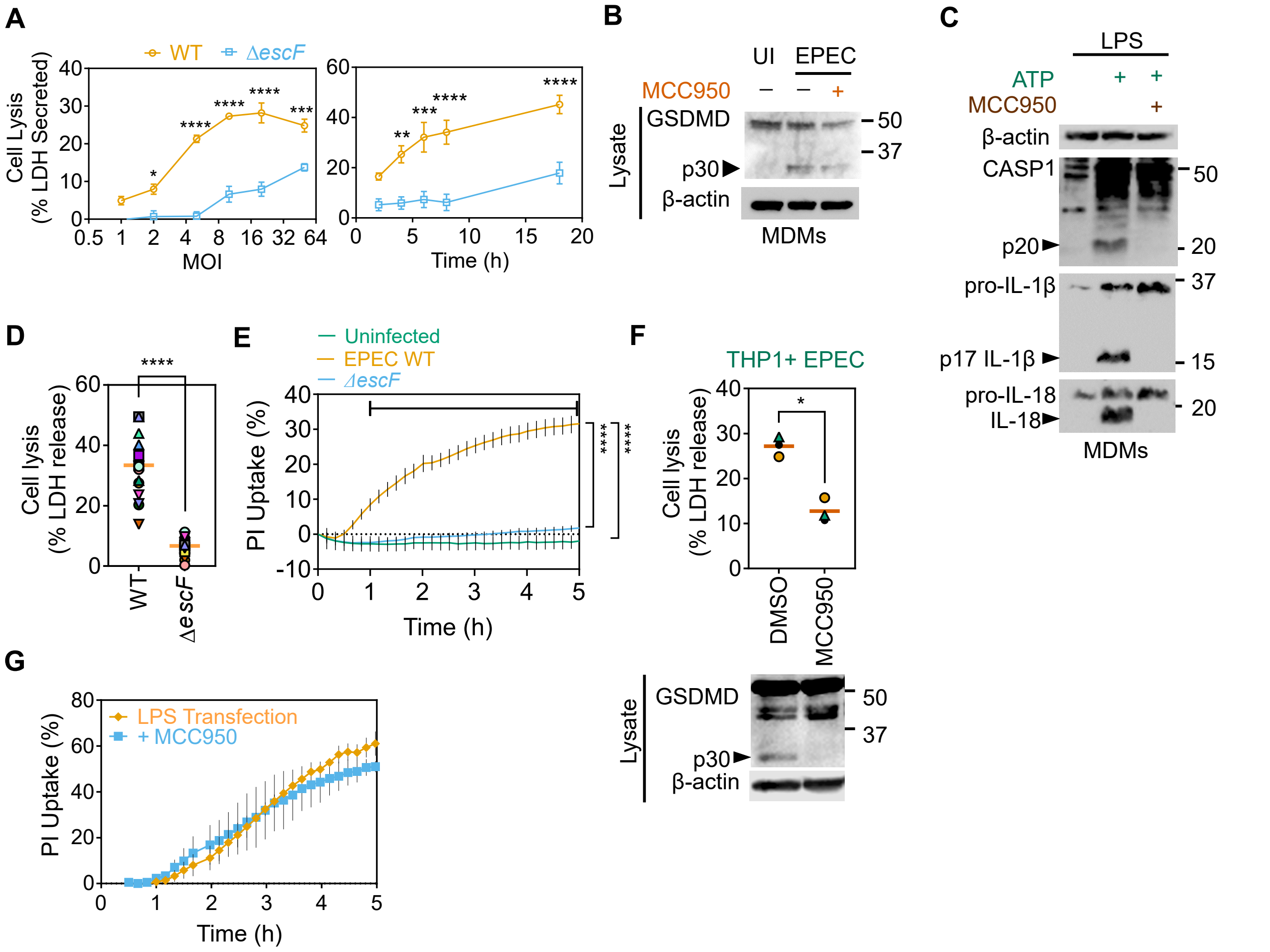

Figure S1. EPEC induces rapid NLRP3-dependent pyroptosis in THP1 cells (related to Figure 1)

(**A**) LDH-release assays from THP1 cells infected with DMEM-primed wildtype (WT) or Δ*escF* EPEC at indicated bacteria:macrophage multiplicity of infection (MOI) for 4 h (left) or at MOI 10 for indicated time (right). Mean±sem from n=3 experiments plotted. * *P<0.05*, ** *P<0.01*, *** *P<0.001*, **** *P<0.0001* by two-way ANOVA for comparisons of EPEC strains at indicated time points.

(**B**) Representative immunoblots from primary MDMs infected with EPEC in the absence (-) or presence of MCC950 (5 µM) for 4 h (*n=3* independent donors).

(**C**) Representative immunoblots from primary MDMs treated with LPS for 3 h and left untreated, or treated with ATP (5 mM; 60 min) in the absence or presence of MCC950. Western blot for β-actin was performed on cell lysates and other proteins on supernatants.

(**D-E**) Cell lysis measured as LDH release (**D**) or PI uptake (**E**) from THP1 cells left uninfected (UI) or infected with indicated wildtype (WT) or Δ*escF* EPEC strains. **** *P<0.0001* by two-tailed Student's t-test in (**D**). **** *P<0.0001* by two-way ANOVA for indicated comparisons after FDR-based correction for multiple comparisons in (**E**).

(**F**) LDH release assay (top) and representative immunoblots (below) from THP1 cells infected with EPEC in the absence (-) or presence of MCC950 (5 µM) for 4 h (*n=3* independent experiments).

(**G**) Real-time PI uptake of THP1 cells transfected with LPS using Lipofectamine 2000. MCC950 was used at 5 µM.

Matching shapes and colours of symbols in graphs in **(D)** and **(F)** denote data from independent experiments. Mean±sem plotted from *n=3* independent experiments in **(E)** and **(G)**. **P<0.05*, **** *P<0.0001* by two-tailed paired Student's *t*-test.

Goddard *et al* Figure S2

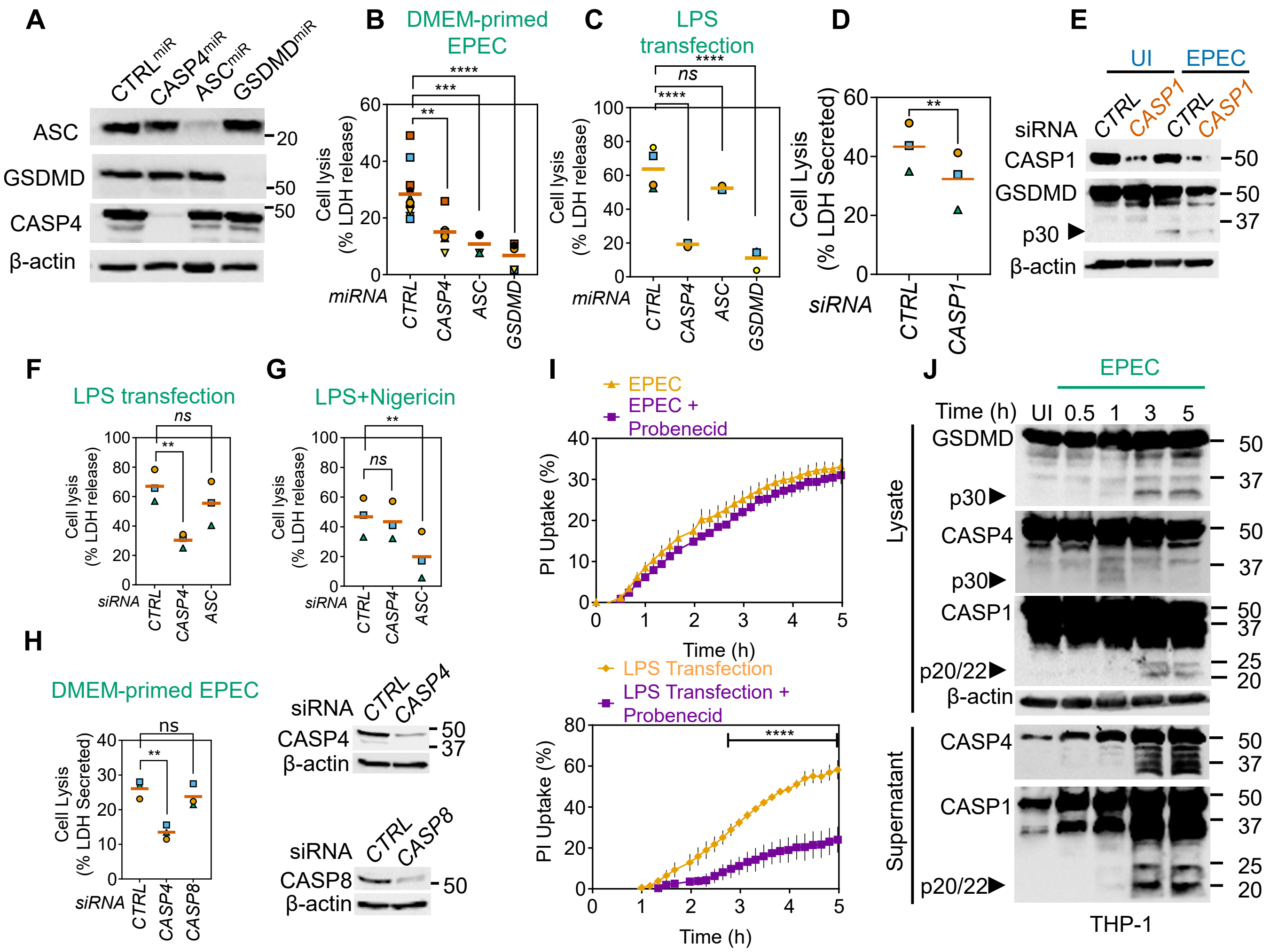

Figure S2. EPEC priming in DMEM alters caspase-4-driven pyroptosis signalling (related to Figures 2-3)
(**A**) Representative immunoblots from THP1 cells stably expressing non-targeting (CTRL) or miRNA30E (miR) against indicated genes showing silencing of protein expression.
(**B**) Cell lysis assay from indicated THP1 miRNA30E-expressing cells infected with DMEM-primed EPEC for 4 h. (*n=4-6* independent experiments)
(**C**) Cell lysis assay from indicated THP1 miRNA30E-expressing cells transfected with LPS for 4 h. (*n=3-4* independent experiments)
(**D-E**) LDH-release assay (**D**) and representative immunoblots of cell lysates (**E**) from THP1 cells transfected with non-targeting control (CTRL) or caspase-1 siRNA for 72 h and infected with EPEC (*n=3* independent experiments). ** *P<0.01* by two-tailed paired Student's *t*-test.
(**F-G**) LDH-release assay from THP1 cells transfected with indicated siRNA for 72 h and transfected with LPS in (**F**) or treated with LPS+nigericin in (**G**) (*n=3* independent experiments).
(**H**) LDH-release assay (left) and representative western blots (right) from THP1 cells transfected with indicated siRNA for 72 h followed by infection with EPEC for 4 h (*n=3* independent experiments).
(**I**) Real-time PI-uptake assay from THP1 cells either infected with DMEM-primed EPEC (top) or transfected with LPS (bottom) in the absence or presence of probenecid (100 μM). Mean±sem from *n=3* independent experiments are plotted. **** *P<0.0001* by two-way ANOVA with FDR-based correction for multiple comparisons.
(**J**) Representative immunoblots of cell lysates or supernatants of THP1 cells left uninfected (UI) or infected with DMEM-primed EPEC for indicated times.
Matching shapes and colours of symbols in graphs in (**B-D, F-H**) denote data from independent experiments. Immunoblots are representative of experiments performed at least three times. ** *P<0.01,* *** *P<0.001,* **** *P<0.0001* for indicated comparisons by one-way ANOVA in **B, C, F-H** and two-way ANOVA in **I**; ns – not significant.

Goddard *et al* Figure S3

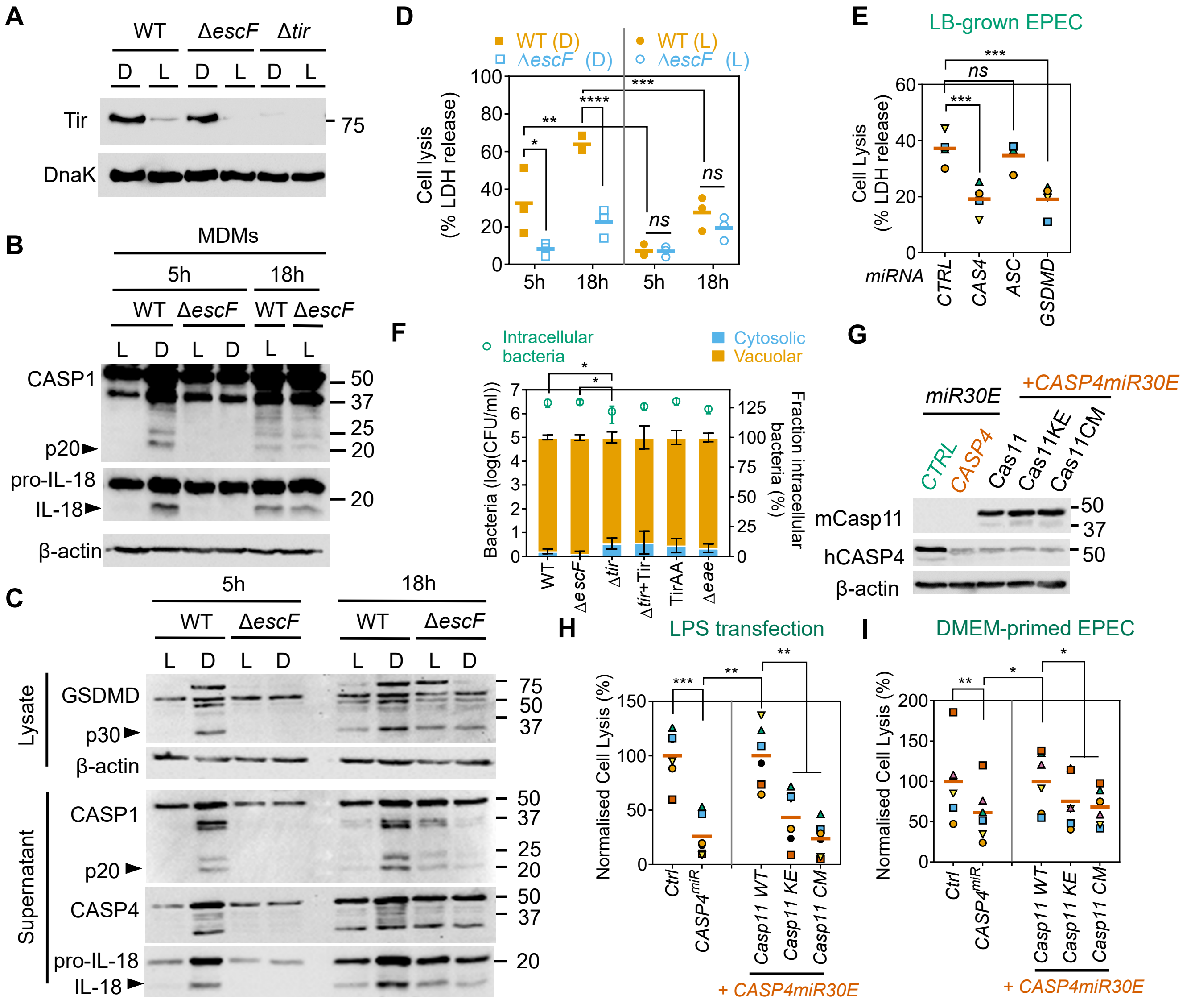

Figure S3. Virulence regulon expression influences EPEC-induced pyroptosis (related to Figures 2-3)
(**A**) Expression of Tir and DnaK (as a house-keeping gene) in indicated strains of EPEC grown with DMEM-priming (D) or in lysogeny broth (L).
(**B-C**) Representative immunoblots from MDMs (**B**) or THP1 (**C**) infected with indicated strains of EPEC grown with DMEM-priming (D) or in LB (L) at 5 h or 18 h post-infection as labelled.
(**D**) LDH release measurements from THP1 cells infected with indicated strains of EPEC (MOI 10) which were DMEM-primed (D) or LB-grown (L) after 5 h or 18 h of infection (*n=3* experiments).
(**E**) Cell lysis assay from indicated THP1 miRNA30E-expressing cells infected with LB-grown EPEC (MOI 10) for 18 h. (*n=4* independent experiments).
(**F**) Total intracellular bacteria (left axis) and fraction of intracellular bacteria (right axis) for indicated strains of EPEC at 4 h post-infection of THP1^{GSDMDmiR} cells. Mean±sem from *n*=4-6 independent experiments are plotted. Mean fractional cytosolic bacteria are similar (4-12 %) for all strains. * *P<0.05* by one-way ANOVA and Tukey's test for indicated comparisons of total intracellular bacterial CFU; means of other strains are not different when compared to WT or Δ*tir*.
(G) Representative immunoblots from THP1 cells stably expressing non-targeting (*CTRL*) or *CASP4*-miRNA30E (miR30E) and *CASP4miR* expressing cells reconstituted with indicated mCaspase-11 variants.
(H-I) Cell lysis assays from indicated THP1 cells transfected with LPS (**H**) or infected with DMEM-primed EPEC (**I**) for 4 h (*n=6* independent experiments).
Immunoblots are representative of 2-3 independent repeats. Matching shapes and colours of symbols in graphs in **E, H** and **I** denote data from independent experiments. * *P<0.05,* ** *P<0.01,* *** *P<0.001,* **** *P<0.0001* for indicated comparisons by one-way ANOVA in **E, F, H, I** and two-way ANOVA in **D**; ns – not significant.

Goddard *et al* Figure S4

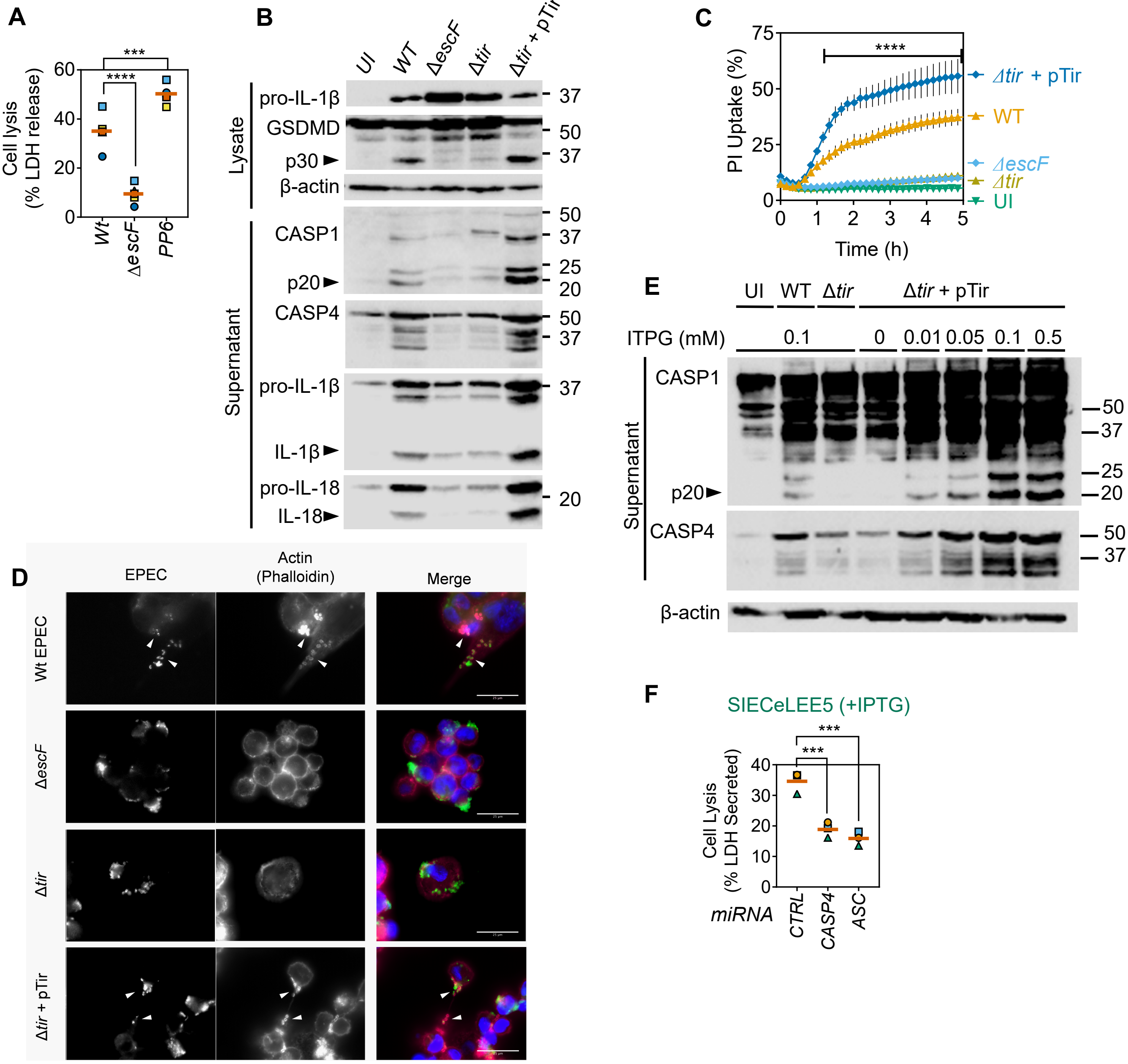

Figure S4. Tir is required for EPEC-induced inflammasome activation (related to Figure 4)

(**A**) LDH release assay from THP1 cells infected with indicated strains of EPEC for 5 h. *n=5* independent experiments. *** *P<0.001,* **** *P<0.0001* by one-way ANOVA. Matching shapes and colours of symbols in graphs denote data from independent experiments.

(**B-C**) Representative immunoblots (**B**) and PI uptake assay (**C**) from THP1 cells infected with indicated EPEC strains. IPTG (0.1 mM) was added 30 min prior to infection to induce Tir expression from pSA10 plasmid (pTir). Mean±sem from *n= 3-6* independent experiments plotted in (**C**). **** *P<0.0001* for comparisons by two-way ANOVA between WT EPEC and other strains at indicated time points after FDR-based correction for multiple comparisons.

(**D**) Representative immunofluorescence microscopy images of THP1 cells infected with indicated EPEC strains for 2 h. Arrowheads indicate actin polymerisation. Scale bar – 25 µm.

(**E**) Representative immunoblots from THP1 cells infected for 4 h with Δ*tir* EPEC expressing Tir from an IPTG-inducible plasmid (pTir). Bacteria were treated with indicated concentrations of IPTG for 30 min before infection.

(**F**) LDH-release assay from THP1 cells expressing *CTRL*, *CASP4* or *ASC* miRNA infected for 4 h with SIECeLEE5 (treated with IPTG). *** *P<0.001* by one-way ANOVA.

Matching shapes and colours of symbols in **A** and **F** denote data from independent experiments.

Immunoblots are representative of experiments performed at least two times.

Goddard *et al* Figure S5

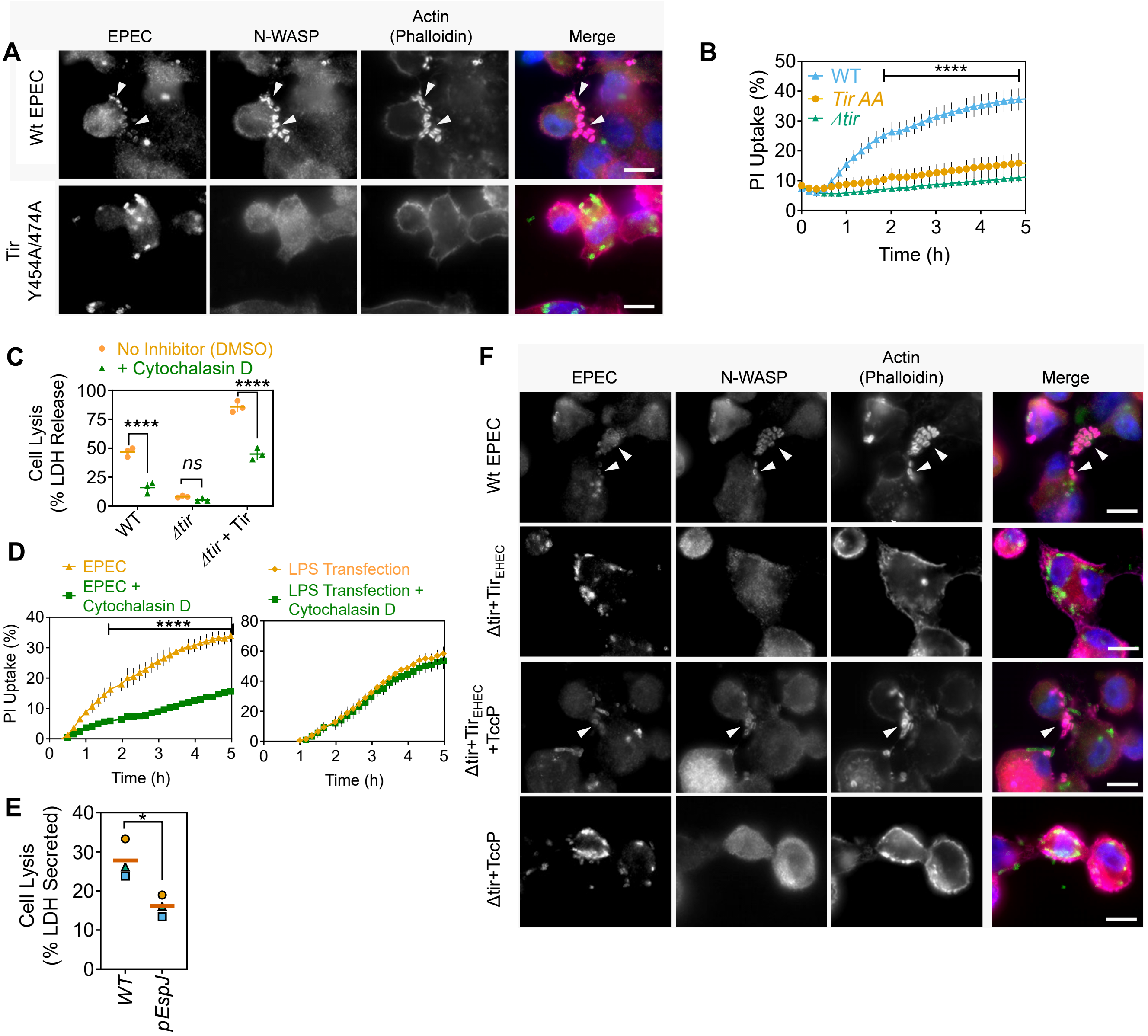

Figure S5. Tir-induced actin polymerisation drives inflammasome activation (related to Figure 5)
(**A**) Representative images from immunofluorescence microscopy of THP1 cells infected with wildtype (Wt) EPEC and EPEC expressing Tir^{AA} (Y454A/Y474A) for 2 h. Arrowheads, bacteria with actin-rich pedestals. Scale bar – 20μm.
(**B**) Real-time PI uptake assay of THP1 cells infected with indicated EPEC strains (mean±sem from *n= 3-6* independent experiments). **** *P<0.0001* by two-way ANOVA for comparisons of WT EPEC with other strains at indicated time points with FDR-based correction for multiple comparisons.
(**C**) Cell lysis measured as LDH release from THP1 cells infected with indicated EPEC strains in the absence or presence of Cytochalasin D (200 nM). *n=4* independent experiments. **** *P<0.0001, ns* – not significant by two-way ANOVA.
(**D**) Real-time PI uptake assay from THP1 cells infected with EPEC (left) or transfected with LPS (right) in the absence or presence of Cytochalasin D (200 nM). Mean±sem from *n=3* independent experiments plotted. **** *P<0.0001* for indicated comparisons by two-way ANOVA with FDR-based correction for multiple comparisons.
(**E**) Cell lysis (LDH assays) from THP1 cells infected with wildtype EPEC (WT) or a strain expressing EspJ on a plasmid (pEspJ) at MOI 10 for 4 h (*n=3* independent experiments). * *P<0.05* by two-tailed Student's *t*-test.
(**F**) Representative images from immunofluorescence microscopy of THP1 cells infected with indicated EPEC strains expressing Tir from EPEC or EHEC, or additionally expressing EHEC TccP. Arrowheads, bacteria with actin-rich pedestals. Scale bar – 20μm.
Matching shapes and colours of symbols in **E** denote data from independent experiments.

Table S1: sequences of SMART Pool siRNAs

siRNA	Source	Cat. No.
siRNA ON-TARGETplus non-targeting	Dharmacon	D-001810-10
- UGGUUUACAUGUCGACUAA -		(D-001810-01)
- UGGUUUACAUGUUGUGUGA -		(D-001810-02)
- UGGUUUACAUGUUUUCUGA -		(D-001810-03)
- UGGUUUACAUGUUUUCCUA -		(D-001810-04)
siRNA CASP4	Dharmacon	L-004404-00
- GGACUAUAGUGUAGAUGUA -		(L-004404-05)
- CAACGUAUGGCAGGACAAA -		(L-004404-06)
- GAACUGUGCAUGAUGAGAA -		(L-004404-07)
- UAACAUAGACCAAAUAUCC -		(L-004404-08)
siRNA ASC (PYCARD)	Dharmacon	L-004378-00
- GGAAGGUCCUGACGGAUGA -		(J-004378-06)
- UCACAAACGUUGAGUGGCU -		(J-004378-07)
- GGCCUGCACUUUAUAGACC -		(J-004378-08)
- CCACCAACCCAAGCAAGAU -		(J-004378-09)
siRNA CASP8	Dharmacon	L-003466-00
- GGACAAAGUUUACCAAAUG -		(J-003466-13)
- GCCCAAACUUCACAGCAUU -		(J-003466-14)
- GAUAAUCAACGACUAUGAA -		(J-003466-15)
- GUCAUGCUCUAUCAGAUUU -		(J-003466-16)
