# Supplementary material for: Enteropathogenic Escherichia coli Stimulates Effector-Driven Rapid Caspase-4 Activation in Human Macrophages
Source: Cell Rep. 2019 Apr 23;27(4):1008–1017.e6. doi: 10.1016/j.celrep.2019.03.100 (PMC6486487; doi:10.1016/j.celrep.2019.03.100)
Supplement: Document S2. Article plus Supplemental Information [file mmc2.pdf]

Cell Reports

Report

Enteropathogenic *Escherichia coli* Stimulates Effector-Driven Rapid Caspase-4 Activation in Human Macrophages

Graphical Abstract

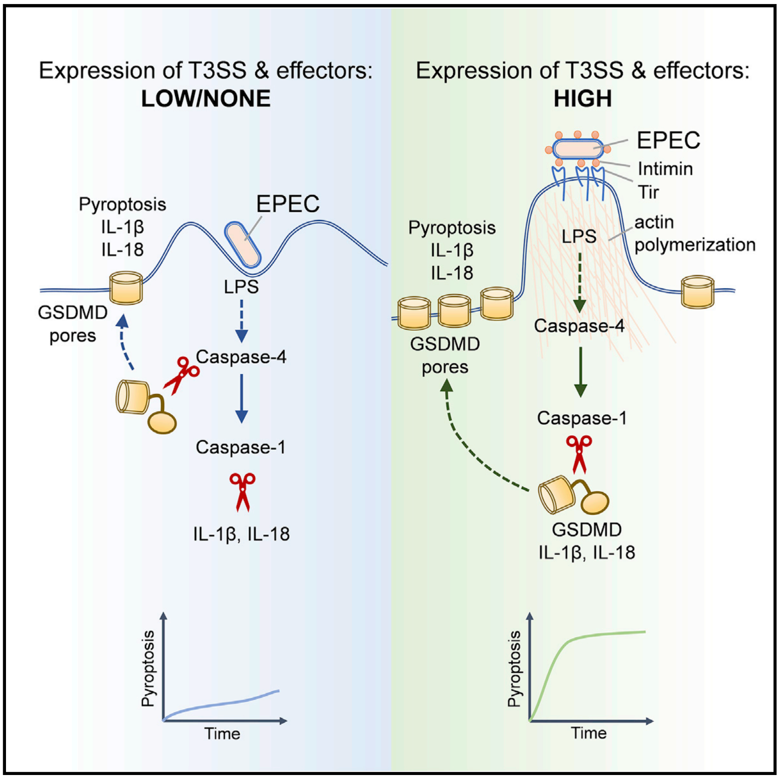

Authors

Philippa J. Goddard,
Julia Sanchez-Garrido,
Sabrina L. Slater, ...,
Luis Ángel Fernández, Gad Frankel,
Avinash R. Shenoy

Correspondence

a.shenoy@imperial.ac.uk

In Brief

Goddard et al. show that enteropathogenic *Escherichia coli* triggers pyroptosis via caspase-4-mediated LPS sensing and actin polymerization driven by the effector Tir and its ligand intimin. The Tir-intimin signaling cascade stimulates mechanistically distinct rapid atypical macrophage pyroptosis and cytokine processing via the NLRP3-caspase-1 inflammasomes.

Highlights

- EPEC bacteria expressing virulence genes induce rapid human macrophage pyroptosis
- Bacterial LPS sensing by caspase-4 activates NLRP3-caspase-1 inflammasomes
- Actin polymerization driven by Tir-intimin signaling promotes pyroptosis
- Caspase-1 mediates cytokine processing and gasdermin D cleavage, leading to pyroptosis

Goddard et al., 2019, Cell Reports *27*, 1008–1017
April 23, 2019 © 2019 The Author(s).
https://doi.org/10.1016/j.celrep.2019.03.100

CellPress

OPEN
ACCESS
CellPress

Cell Reports
Report

Enteropathogenic *Escherichia coli* Stimulates Effector-Driven Rapid Caspase-4 Activation in Human Macrophages

Philippa J. Goddard,[1,2] Julia Sanchez-Garrido,[2] Sabrina L. Slater,[1] Mohini Kalyan,[2] David Ruano-Gallego,[3] Olivier Marchès,[1,4] Luis Ángel Fernández,[3] Gad Frankel,[1] and Avinash R. Shenoy[2,5,*]
[1]Department of Life Sciences, Medical Research Council Centre for Molecular Bacteriology & Infection, Imperial College London, London, UK
[2]Department of Medicine, Medical Research Council Centre for Molecular Bacteriology & Infection, Imperial College London, London, UK
[3]Department of Microbial Biotechnology, Centro Nacional de Biotecnología, Consejo Superior de Investigaciones Científicas (CSIC), Madrid, Spain
[4]Present address: Blizard Institute, Barts and the London School of Medicine and Dentistry, Queen Mary University of London, London, UK
[5]Lead Contact
*Correspondence: a.shenoy@imperial.ac.uk
https://doi.org/10.1016/j.celrep.2019.03.100

SUMMARY

Microbial infections can stimulate the assembly of inflammasomes, which activate caspase-1. The gastrointestinal pathogen enteropathogenic *Escherichia coli* (EPEC) causes localized actin polymerization in host cells. Actin polymerization requires the binding of the bacterial adhesin intimin to Tir, which is delivered to host cells via a type 3 secretion system (T3SS). We show that EPEC induces T3SS-dependent rapid non-canonical NLRP3 inflammasome activation in human macrophages. Notably, caspase-4 activation by EPEC triggers pyroptosis and cytokine processing through the NLRP3-caspase-1 inflammasome. Mechanistically, caspase-4 activation requires the detection of LPS and EPEC-induced actin polymerization, either via Tir tyrosine phosphorylation and the phosphotyrosine-binding adaptor NCK or Tir and the NCK-mimicking effector TccP. An engineered *E. coli* K12 could reconstitute Tir-intimin signaling, which is necessary and sufficient for inflammasome activation, ruling out the involvement of other virulence factors. Our studies reveal a crosstalk between caspase-4 and caspase-1 that is cooperatively stimulated by LPS and effector-driven actin polymerization.

INTRODUCTION

The human gastrointestinal pathogens enteropathogenic *Escherichia coli* (EPEC) and enterohemorrhagic *E. coli* (EHEC) colonize the gut mucosa while forming attaching and effacing (A/E) lesions, which are characterized by the effacement of the brush border microvilli and intimate bacterial attachment to the apical surface of intestinal epithelial cells (IECs) (Frankel et al., 1998). Intimate attachment is mediated by the binding of intimin, a bacterial outer membrane adhesin, to the translocated intimin receptor (Tir), which is delivered into mammalian cells via a type III secretion system (T3SS) injectisome (Kenny et al., 1997). The T3SS is encoded by four operons, i.e., locus for enterocyte effacement (LEE) 1–4, and the monocistronic *escD* gene within the LEE pathogenicity island (McDaniel et al., 1995; Elliott et al., 1998), and translocates multiple LEE-encoded (e.g., Tir, Map, EspG) and non-LEE- encoded (e.g., EspJ, NleA-F, TccP) effectors that manipulate signaling in the host cell (Wong et al., 2011; Pearson et al., 2016; Shenoy et al., 2018). Expression of the T3SS and effector genes can be induced by growing EPEC/EHEC in low-glucose DMEM (DMEM priming) *in vitro* (Rosenshine et al., 1996; Abe et al., 2002; Furniss and Clements, 2017).

The clustering of Tir_{EPEC} by intimin induces the phosphorylation of Tyr474 in the C terminus of Tir by redundant non-receptor tyrosine kinases (e.g., Src, ABL) (Wong et al., 2011; Pearson et al., 2016). The Src homology domain 2- and 3- (SH2 and SH3) containing adaptor NCK interacts with phosphorylated tyrosine residues in Tir and recruits N-WASP (neural Wiskott-Aldrich syndrome protein), which activates the ARP2/3 (actin-related protein-2/3) complex leading to the formation of actin-rich pedestal-like structures at sites of bacterial attachment. Although Tir is conserved in all A/E pathogens, ARP2/3 activation and actin polymerization by Tir_{EHEC} (e.g., O157:H7) requires TccP (Tir-cytoskeleton coupling protein) (Garmendia et al., 2004; Campellone et al., 2004), which is recruited by IRTKS (insulin receptor tyrosine kinase substrate) or IRSp53 (insulin receptor substrate p53) adaptors via their interaction with the conserved NPY motif in Tir (Vingadassalom et al., 2009; Weiss et al., 2009; Lai et al., 2013). TccP structurally mimics the autoinhibitory element within N-WASP, leading to ARP2/3-dependent phosphotyrosine-independent actin polymerization (Frankel and Phillips, 2008). The physiological role of Tir-induced actin polymerization is poorly understood.

Macrophages can promote host defense by sensing and responding to infection via inflammasomes, which are signaling platforms that activate caspase-1 (Eldridge and Shenoy, 2015; Broz and Dixit, 2016). A/E pathogen-associated molecules, including lipopolysaccharides (LPS), nucleic acids, and T3SS inner rod and needle proteins, can activate caspase-1 via the NOD

 © 2019 The Author(s).
This is an open access article under the CC BY license (http://creativecommons.org/licenses/by/4.0/).

leucine-rich repeat proteins (NLRs) and the adaptor protein ASC (Rathinam et al., 2012; Kailasan Vanaja et al., 2014; Vanaja et al., 2016; Zhao et al., 2011; Yang et al., 2013; Kayagaki et al., 2013). The activation of caspase-1 in macrophages leads to the proteolytic maturation of pro-interleukin (IL)-1β and pro-IL-18 and pyroptosis through the proteolysis of gasdermin-D (GSDMD) (Broz and Dixit, 2016), which together promote immunity against infection (Liu et al., 2012; Nordlander et al., 2014; Song-Zhao et al., 2014).

NLRP3 (NOD, leucine-rich repeat and Pyrin domain-containing protein 3) inflammasome assembly is stimulated by the loss of cytosolic K^+, which can occur via two broadly distinct mechanisms. Canonical NLRP3 activation involves K^+ efflux by the opening of P2X7 channels by its ligand ATP or bacterial ionophore toxins (e.g., nigericin) (Broz and Dixit, 2016). The non-canonical NLRP3 pathway involves the activation of caspase-11 in mouse cells and caspase-4 or caspase-5 in human cells by cytosolic LPS, which leads to the cleavage of GSDMD, efflux of K^+, and pyroptosis (Kayagaki et al., 2015). LPS sensing also leads to pro-IL-1β and pro-IL-18 processing via caspase-1 activation by the NLRP3-ASC inflammasome (Kayagaki et al., 2015; Shi et al., 2015). Moreover, activation of caspase-11 by LPS can lead to the cleavage of pannexin-1 channels, resulting in pyroptosis and the release of ATP, which can also activate NLRP3 (Yang et al., 2015).

EHEC and the mouse A/E pathogen *Citrobacter rodentium* grown without DMEM priming (i.e., bacteria poorly expressing the virulence regulon) stimulate inflammasomes similarly to non-pathogenic *E. coli* K12, resulting in non-canonical NLRP3 activation in mouse macrophages at 10–18 h post-infection (Rathinam et al., 2012; Gurung et al., 2012; Vanaja et al., 2016). This is also similar to the pathway induced by direct delivery of LPS into the cytosol (e.g., by transfection). Whether EPEC infection activates the inflammasome in human macrophages and the impact of virulence genes on this pathway is presently unknown.

In this study, we investigated inflammasome activation in primary human monocyte-derived macrophages (MDMs) infected with EPEC expressing the virulence regulon. We found an essential role for caspase-4 in rapid caspase-1 activation via NLRP3 in response to virulent EPEC. Tir-mediated actin polymerization was indispensable for inflammasome activation in a manner that markedly differed from that induced by the transfection of cytosolic LPS. Our findings establish a crosstalk between caspase-4 and caspase-1 during natural infection by virulent EPEC.

RESULTS

EPEC Induces T3SS-Dependent Activation of NLRP3 in Human Macrophages

We investigated whether DMEM-primed EPEC activated the inflammasome in primary human $CD14^+$ MDMs and phorbol 12-myristate 13-acetate (PMA)-differentiated THP1 cells by measuring pyroptotic cell death (release of lactate dehydrogenase [LDH]), uptake of propidium iodide (PI) dye, and immunoblots for caspase-1 and its substrates. Cells infected at a multiplicity of infection (MOI) of 10 for 4–5 h underwent pyroptotic death within 4 h of infection in a T3SS-dependent manner (Figures 1A and S1A). Immunoblots confirmed the presence of active caspase-1 p20 and proteolytic processing of pro-IL-18 upon infection with wild-type (WT) EPEC but not the T3SS-deficient Δ*escF* mutant (Figure 1A). To test whether NLRP3 was required for EPEC-induced caspase-1 activation, we used the NLRP3-specific inhibitor MCC950 (Coll et al., 2015; Gaidt et al., 2017). This revealed that MCC950 treatment blocked EPEC-induced LDH release (~85% reduction), caspase-1 activation, and reduced GSDMD proteolysis (Figures 1B, 1C, and S1B). MCC950 also inhibited LPS plus ATP-mediated caspase-1 activation as a positive control of canonical NLRP3 activation (Figure S1C).

Like primary MDMs, PMA-differentiated THP1 cells also underwent T3SS-dependent rapid pyroptosis as observed by real-time PI uptake and immunoblots, which showed the proteolysis of caspase-1, the pyroptosis-associated substrate GSDMD, and pro-IL-1β and pro-IL-18 cytokine precursors (Figures 1D, S1D, and S1E). Moreover, the treatment of THP1 cells with MCC950 also blocked pyroptosis and caspase-1 activation by EPEC (Figures 1E, 1F, and S1F). As expected, pyroptosis induced by LPS transfection, as a positive control for non-canonical caspase-4-dependent pyroptosis, was not inhibited by MCC950 (Figure S1G). We therefore concluded that EPEC activates rapid cytokine processing and pyroptosis via the NLRP3-caspase-1 inflammasome in primary MDMs and THP1 macrophages in a T3SS-dependent manner.

NLRP3-Caspase-1 Activation via Caspase-4 Drives EPEC-Induced Pyroptosis

To obtain independent support for the involvement of NLRP3-ASC-caspase-1 and pyroptosis in EPEC-induced cell death, we used RNAi. Stable silencing of *GSDMD* or *ASC* ($THP1^{GSDMDmiR}$ or $THP1^{ASCmiR}$ cells) attenuated pyroptosis induced by EPEC as measured by PI uptake and LDH release assays (Figures 2A, S2A, and S2B). As a control, we verified that LPS transfection of these cells triggered ASC-independent and GSDMD-dependent pyroptosis and ASC- and GSDMD-dependent proteolytic processing of caspase-1 (Figures 2B, 2C, and S2C). In agreement with MCC950 treatment, EPEC-induced proteolysis of GSDMD was blocked by the silencing of ASC, further pointing toward a role for caspase-1 in the process (Figure 2D). In agreement with these findings, silencing *CASP1* expression reduced pyroptosis and GSDMD cleavage in response to EPEC infection (Figures S2D and S2E). These results indicated that the NLRP3-ASC-caspase-1 inflammasome plays a major role in GSDMD processing and pyroptosis during EPEC infection.

Because caspase-4 is involved in sensing Gram-negative pathogens such as *Salmonella, Legionella*, and *Francisella* in primary human MDMs (Casson et al., 2015; Lagrange et al., 2018), we next examined its involvement upstream of the NLRP3-ASC-caspase-1 inflammasome during EPEC infection (see schematics in Figures 2C and 2E). Stable silencing of caspase-4 ($THP1^{CASP4miR}$) markedly attenuated pyroptosis (Figures 2A and S2B) and caspase-1 activation (Figure 2E). Independent validation was obtained with small interfering RNA (siRNA) against caspase-4, which showed it was required for pyroptosis and IL-1β release as measured by ELISA from EPEC-infected macrophages (Figure 3A); as expected, caspase-4 was required

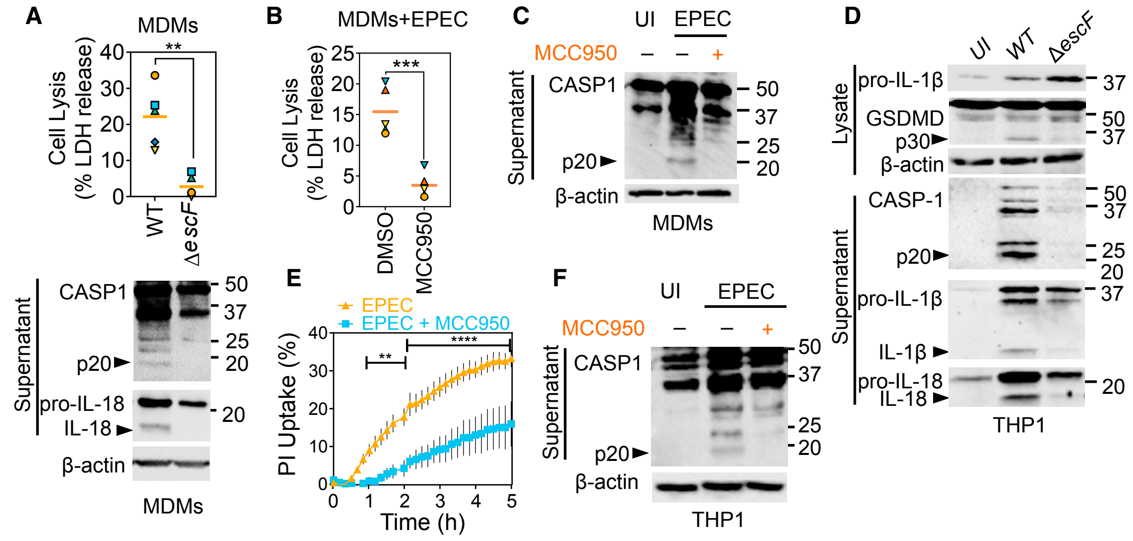

Figure 1. EPEC Induces Rapid, NLRP3-Dependent Pyroptosis and Cytokine Processing in Human Monocyte-Derived Macrophages and THP1 Cells

(A) Primary human monocyte-derived macrophage (MDMs) were infected with DMEM-primed wild-type (WT) or T3SS-deficient Δ*escF* EPEC for 4 h. Pyroptosis measured by lactate dehydrogenase (LDH) release (n = 6 independent repeats from four donors) is plotted on top in (A) and representative immunoblots for indicated proteins are shown below.

(B and C) Primary MDMs were left uninfected (UI) or infected with WT EPEC without or with MCC950 (5 μM). LDH release assay (B) and representative immunoblots (C) are shown (n = 5 independent repeats from four donors).

(D–F) THP1 cells were left UI or infected with DMEM-primed indicated strains of EPEC (WT or T3SS-deficient Δ*escF*) for 4 h (D and F) or up to 5 h (E). The graph in (E) shows real-time propidium iodide (PI) uptake (means ± SEMs; n = 3 independent experiments). MCC950 was used at 5 μM. **p < 0.01, ****p < 0.0001 by two-way ANOVA with false discovery rate (FDR)-based correction for multiple comparisons.

The matching shapes and colors of symbols in graphs in (A) and (B) denote data from independent donors and/or experiments. Immunoblots (A, C, D, and F) are representative of experiments performed at least three times. **p < 0.01, ***p < 0.001 by two-tailed paired Student's t test.

for pyroptosis induced by cytosolic LPS, but not during treatment with LPS plus nigericin (Figures S2F and S2G). *CASP4* silencing in primary MDMs also decreased pyroptosis and IL-1β release upon EPEC infection and LPS transfection (Figure 3B). *CASP8* silencing had no impact on EPEC-induced pyroptosis (Figure S2H). Mechanistically, GSDMD and pannexin-1 have been implicated in the non-canonical activation of caspase-1 (schematic in Figure 2C) (Kayagaki et al., 2015; Yang et al., 2015). We found that GSDMD silencing, but not the inhibition of pannexin-1 channels, abrogated pyroptosis and caspase-1 activation in response to EPEC infection (Figures 2A, 2D, and S2I). Consistent with a temporally early role of caspase-4 in EPEC-induced pyroptosis, immunoblots showed caspase-4 activation in cell lysates within 1 h of infection, whereas cleaved caspase-1 and GSDMD proteins were detected at 3 and 5 h post-infection (Figure S2J). These results revealed a surprising role for GSDMD and caspase-4 upstream of cytokine processing and pyroptosis induced by caspase-1 during EPEC infection.

Non-pathogenic *E. coli* and lysogeny broth (LB)-grown EHEC activate late caspase-4-dependent pyroptosis in mouse macrophages (Kayagaki et al., 2015; Rathinam et al., 2012; Vanaja et al., 2016). We found that infection by LB-grown EPEC caused weaker and delayed inflammasome activation in primary MDMs and THP1 cells, which markedly contrasted DMEM-primed bacteria (Figures S3A–S3D). Mechanistically, pyroptosis induced by LB-grown EPEC was kinetically delayed, independent of the T3SS, ASC independent, and GSDMD dependent (Figure S3E). In particular, DMEM-primed bacteria induced ~5-fold higher pyroptosis than LB-grown bacteria at 5 h post-infection. Furthermore, LB-grown WT and Δ*escF* induced comparable levels of pyroptosis at 5 and 18 h post-infection (Figures S3A–S3D). These results suggested that DMEM priming of EPEC not only accelerated pyroptosis but also altered the inflammasome signaling mechanisms that drive pyroptosis in host cells. We concluded that EPEC expressing virulence genes upregulated by DMEM priming (e.g., the LEE and non-LEE regulons) provoked rapid atypical inflammasome signaling, in which caspase-4 mediated NLRP3-caspase-1 activation, but did not directly induce pyroptosis on its own.

LPS Sensing by Caspase-4 Promotes EPEC-Induced Inflammasome Activation

LPS from intracellular cytosolic and/or vacuolar Gram-negative pathogens are a potent activator of caspase-4. We therefore investigated whether reduced pyroptosis upon infection with Δ*escF* was due to its reduced internalization or cytosolic escape as compared to WT EPEC. To this end, we performed antibiotic-protection assays using gentamicin- (which kills extracellular bacteria) and chloroquine- (which kills vacuolar bacteria) protection assays (Klein et al., 2017; Thurston et al., 2016) in THP1^{GSDMDmiR} cells, which do not lyse during infection. This revealed similar numbers of intracellular WT and Δ*escF* EPEC, most of which were vacuolar, and only ~2.5%–5% were

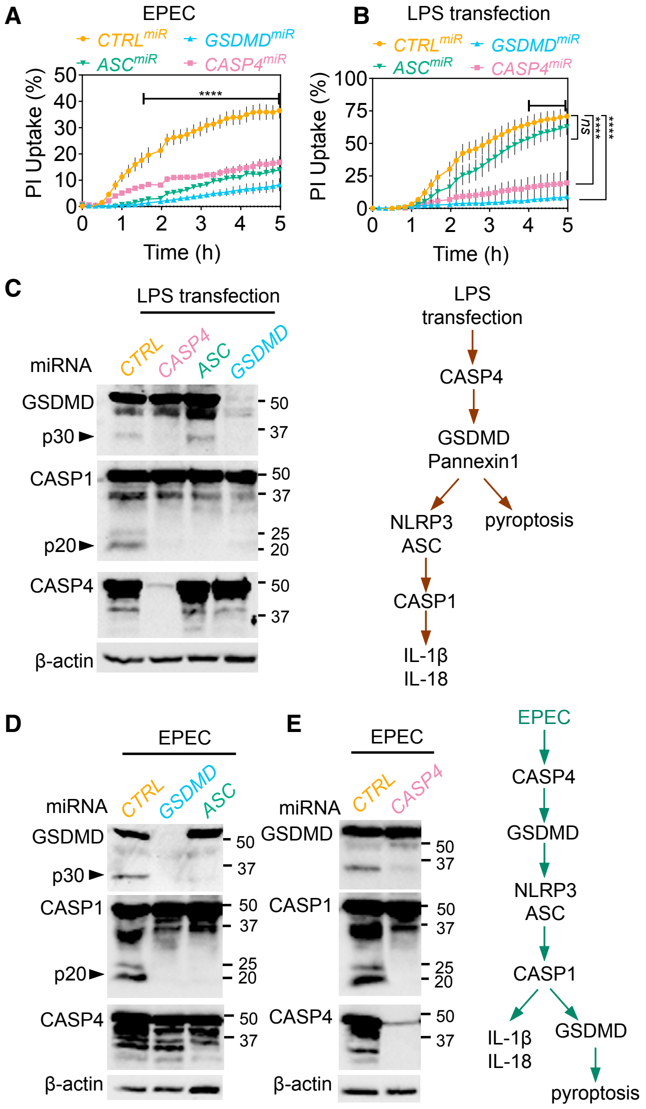

Figure 2. EPEC-Induced Caspase-1 Activation, Cytokine Processing, and Pyroptosis Requires Caspase-4 and GSDMD

(A and B) Real-time PI-uptake assays from THP1 cells stably expressing non-targeting (*CTRL*) or miRNA30E against the indicated genes. THP1 cells were infected with EPEC for indicated times (A) or transfected with LPS using lipofectamine 2000 (B). Means ± SEMs from n = 3 independent experiments are shown. ****$p < 0.0001$ by two-way ANOVA with FDR-based correction for multiple comparisons for indicated comparisons between $CTRL^{miR}$ and others.

(C–E) Representative immunoblots from THP1 cells stably expressing non-targeting (*CTRL*) or miRNA30E against the indicated genes transfected with LPS for 4 h (C) or infected with EPEC for 4 h (D and E). Pooled supernatants and lysates were used for immunoblots. Schematics in (C) and (E) show caspase-4-dependent inflammasome signaling by transfected LPS and EPEC, respectively.

Data in (C)–(E) are representative of experiments performed at least three times.

cytosolic (Figure S3F). These findings suggested that the differential internalization or vacuolar escape was not responsible for the difference in pyroptosis induced by WT and Δ*escF* strains.

While human caspase-4 is activated by the direct binding of LPS in the caspase activation and recruitment domain (CARD), the LPS-binding site in mouse caspase-11 is better defined, and the mutation of three positively charged (lysine) residues in the CARD to glutamate ($Cas11^{KE}$) abrogates LPS binding and non-canonical inflammasome activation (Shi et al., 2015). We reconstituted $THP1^{CASP4miR}$ cells with mouse caspase-11, the LPS-binding mutant ($Cas11^{KE}$) or the catalytically inactive mutant ($Cas11^{CM}$) (Figure S3G). In agreement with previous reports, LPS transfection confirmed that mouse caspase-11, but not $Cas11^{KE}$ or $Cas11^{CM}$, can substitute for human caspase-4 in this setting (Figure S3H) (Shi et al., 2015). Similarly, the expression of caspase-11, but not $Cas11^{KE}$ or $Cas11^{CM}$, also restored rapid pyroptosis in $THP1^{CASP4miR}$ cells infected with EPEC (Figure S3I). These results suggested that EPEC induces T3SS-, LPS-, and caspase-4-dependent inflammasome activation in human macrophages that requires caspase-1 for both pyroptosis and cytokine processing.

Tir Is Essential for Inflammasome Activation by EPEC

We investigated which T3SS effector or effectors were required for inflammasome activation. Consistent with the roles of NleA/EspI (Yen et al., 2015) and NleF (Pallett et al., 2017) in suppressing inflammasome responses, pyroptosis induced by the ΔPP6 strain (which lacks NleA/EspI, NleF, NleH, and EspO) was higher as compared to WT EPEC (Figure S4A). Deletion of other non-LEE pathogenicity islands did not markedly affect pyroptosis in THP1 cells (data not shown), leading us to assess effectors encoded within the LEE. We particularly focused on Tir, which is the dominant LEE-encoded effector. Deletion of Tir attenuated pyroptosis and the proteolysis of GSDMD, caspase-1, pro-IL-1β, and pro-IL-18 in primary MDMs (Figures 4A and 4B) and THP1 cells (Figures 4C, S4B, and S4C). Immunofluorescence microscopy of $THP1^{ASC\text{-}mRFP}$ cells infected with EPEC revealed severely reduced ASC inflammasome foci formation by the Δ*escF* and Δ*tir* strains (Figures 4D and 4E). As expected, actin-rich pedestals were not observed upon the deletion of *escF* or *tir* (Figure S4D). Actin polymerization and inflammasome activation were restored upon complementation of the Δ*tir* strain by ectopic expression of Tir from an isopropyl β-D-1-thiogalactopyranoside (IPTG)-inducible promoter (pTir; Figures 4A–4E and S4E). Furthermore, higher Tir abundance, induced by increasing IPTG concentrations, correlated with elevated pyroptosis (Figure 4F) and proteolysis of caspase-1 (Figure S4E).

We next investigated whether Tir alone was necessary and sufficient for inflammasome activation. To this end, we used the synthetic injector *E. coli* K12 (SIEC) strain, which contains chromosomally integrated T3SS genes under IPTG-inducible promoters (Ruano-Gallego et al., 2015). The SIEC with engineered LEE5 (SIECeLEE5), which additionally expresses Tir, its chaperone CesT, and intimin, efficiently translocates Tir and triggers intimin-dependent actin pedestals (Ruano-Gallego et al., 2015). Similar to WT EPEC, infection of THP1 cells with SIECeLEE5 induced rapid pyroptosis, which was markedly diminished in the absence of eLEE5 (SIEC strain) or the lack of a functional T3SS due to the deletion of the promoter of LEE1 (SIECΔp1) (Figure 4G). Pyroptosis induced by IPTG-treated SIEC bacteria (~20% ± 10%; Figure 4G) was comparable to SIECΔp1 (~8% ± 4%; Figure 4G), which ruled out a major role for NLRC4 (NOD, leucine-rich repeat and CARD-containing

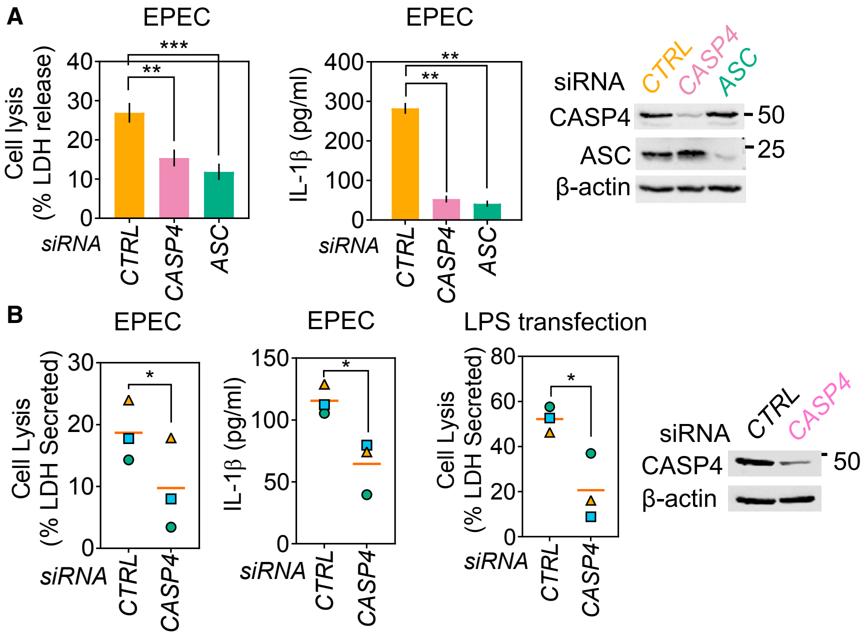

Figure 3. EPEC Induces Caspase-4- and Caspase-1-Dependent Pyroptosis and Cytokine Processing in Human Macrophages

(A) Quantification of pyroptosis by LDH release assay and IL-1β by ELISA from THP1 cells transfected with non-targeting (*CTRL*) or the indicated siRNA and infected with EPEC for 4 h. Representative immunoblots for silencing of caspase-4 and ASC are shown at right. Graphs show LDH release (means ± SEMs from n = 5 independent experiments) and IL-1β release by ELISA (means ± SDs from n = 2 experiments). $^{**}p < 0.01$, $^{***}p < 0.001$ by one-way ANOVA.

(B) Pyroptosis as measured by LDH release and IL-1β quantification by ELISA from primary MDMs transfected with *CTRL* or *CASP4* siRNA and then infected with EPEC or transfected with LPS as labeled. Matching shapes and colors of symbols in graphs represent n = 3 independent donors. $^{*}p < 0.05$ by two-tailed Student's t test.

protein 4) inflammasomes that detect flagellin and T3SS structural proteins. EPEC flagellin and needle (EscF) and rod (EscI) proteins are poor NLRC4 inflammasome activators in human macrophages (Yang et al., 2013; Zhao et al., 2011). Furthermore, similarly to EPEC, IPTG-treated SIECeLEE5 stimulated atypical pyroptosis in a caspase-4 and ASC-dependent manner (Figure S4F). These experiments suggested that intimin and Tir were necessary and sufficient for atypical inflammasome activation in human macrophages.

Tir-Induced Actin Polymerization Is Required for Inflammasome Activation

To test the hypothesis that signaling induced downstream of Tir clustering by its ligand intimin was required for inflammasome activation, we infected macrophages with EPEC expressing Tir Y474A/Y454A (Tir^{AA}, which neither undergoes phosphorylation nor activates N-WASP) (Wong et al., 2012) or the Δ*eae* mutant (which cannot activate Tir as it lacks intimin) (Marchès et al., 2008) (schematic in Figures 5A and S5A). Supporting our hypothesis, EPEC expressing Tir^{AA} and the Δ*eae* strain induced little pyroptosis or inflammasome activation in primary MDMs (~77% reduction; Figures 5B and 5C). Pyroptosis by these strains was similarly attenuated in THP1 cells (~75% reduction; Figures 5D, 5E, and S5B), even though both strains were internalized to levels that are similar to those in WT bacteria (Figure S3F). Treatment with cytochalasin D, which inhibits actin polymerization, impaired pyroptosis induced by EPEC; LPS transfection-induced pyroptosis was not affected by cytochalasin D and served as a negative control (Figures S5C and S5D). In addition, an EPEC strain overexpressing the ADP-ribosyltransferase EspJ, which inactivates non-receptor tyrosine kinases and suppresses Tir signaling (Pollard et al., 2018; Young et al., 2014), also induced less pyroptosis (Figure S5E). These findings pointed to an essential role for Tir-driven actin polymerization in inflammasome activation.

Unlike EPEC, EHEC triggers the formation of actin-rich pedestals by translocating Tir and TccP (schematic in Figure 5A) (Garmendia et al., 2004; Campellone et al., 2004). Complementing the EPEC Δ*tir* mutant with a plasmid encoding Tir_{EHEC} could not restore actin polymerization due to the absence of TccP in EPEC (Figure S5F). The EPECΔ$tir^{+}$$Tir_{EHEC}$ strain also failed to activate inflammasomes (Figures 5F and 5G). In contrast, co-expression of Tir_{EHEC} and TccP in EPECΔ*tir* fully restored actin polymerization and inflammasome activation as quantified by pyroptosis assays and immunoblot analyses of caspase-1 and pro-IL-1β (Figures 5F, 5G, and S5F). Tir-driven actin polymerization, rather than Tir translocation alone or other effectors, is involved in rapid atypical non-canonical inflammasome activation in human macrophages.

DISCUSSION

In this study, we have shown that the infection of human macrophages with EPEC triggers an atypical caspase-4-dependent NLRP3 signaling pathway, which is distinct from that stimulated by LPS transfection or infection by non-pathogenic *E. coli*, and requires signaling downstream of the effector Tir delivered by the pathogen into host cells. Recent studies on inflammasome signaling in human myeloid cells have uncovered surprisingly broad roles for NLRP3, for example, in the detection of cytosolic DNA, which contrasts its roles in mouse macrophages (Gaidt et al., 2017). Similarly, differences have been reported between human and mouse NLRC4 signaling pathways (Zhao and Shao, 2015; Gaidt et al., 2016). Our studies on primary human MDMs support a role for NLRP3 in detecting EPEC infection, which we demonstrate is also conserved in THP1 cells. Atypical signaling (schematic in Figure 2E) involved the combined roles of caspase-4 and GSDMD in promoting NLRP3 activation, and pyroptotic cell death required the activities of both caspase-1 and caspase-4. The NLRP3-ASC-caspase-1 inflammasome rapidly

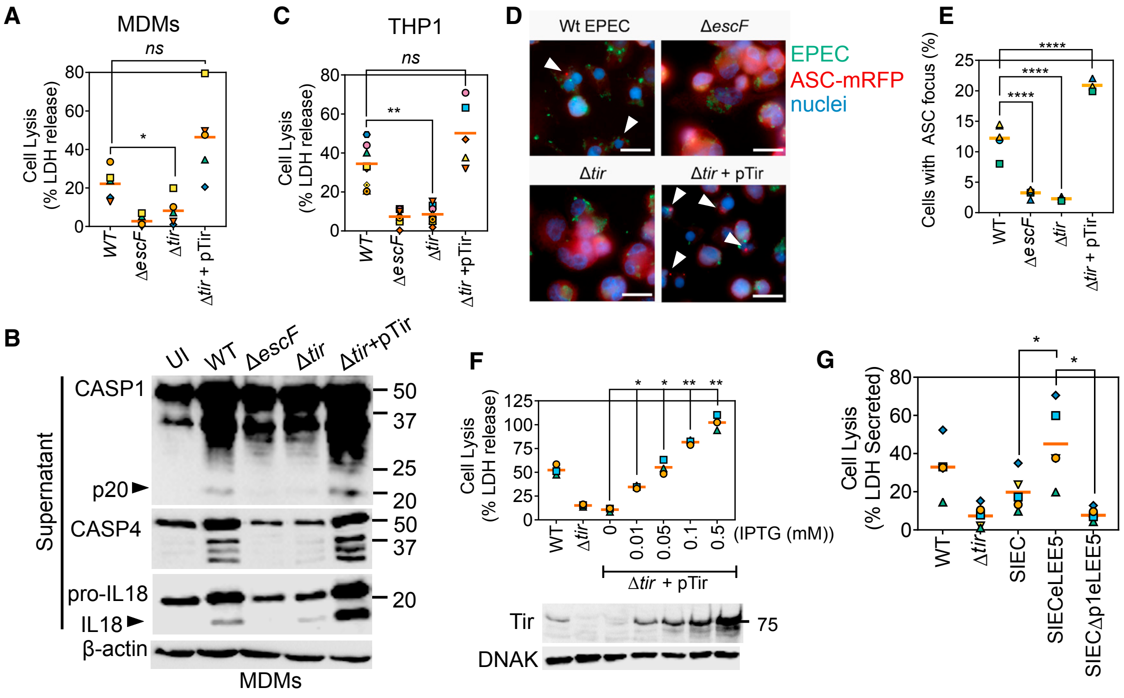

Figure 4. Tir Is Essential for EPEC-Induced Pyroptosis and Cytokine Processing

(A and B) LDH release assays (A) and representative immunoblots (B) from primary human MDMs infected with the indicated EPEC strains for 4 h. IPTG (0.1 mM) was used to induce the expression of pTir. Graph in (A) is from n = 5 independent experiments from four donors. Cell lysates were used for β-actin detection and supernatants for caspase-1, caspase-4, and IL-18.

(C) Quantification of cell lysis (LDH release assay) from THP1 cells infected with the indicated EPEC strains for 4 h (n = 5 independent experiments).

(D) Representative images from immunofluorescence microscopy of THP1$^{ASC\text{-}mRFP}$ cells (red) with the indicated EPEC strains stained with antibody against EPEC (green) and Hoechst nuclear dye (blue) showing re-localization of ASC into foci (arrowheads). Scale bar, 20 μm.

(E) Quantification of ASC specks in experiments described in (D) from n = 3–5 independent experiments.

(F) THP1 cells were infected for 4 h with Δ*tir* EPEC expressing Tir from an IPTG-inducible plasmid (pTir). Bacteria were treated with the indicated concentrations of IPTG for 30 min before infection. LDH release (n = 3 experiments) and representative immunoblots (bottom) for Tir and DnaK are shown.

(G) LDH release assay from THP1 cells infected for 4 h with MOI 10 of synthetic injector *E. coli* (SIEC) strains or WT or Δ*tir* EPEC, as indicated. SIEC strains were treated with IPTG for 30 min before infection to induce the expression of LEE operons (n = 5 independent experiments).

The matching shapes and colors of the symbols in the graphs in (A), (C), and (E)–(G) denote data from independent donors and/or experiments. Immunoblots represent two to three independent experiments. *p < 0.05, **p < 0.01, and ****p < 0.0001 by one-way ANOVA.

processed cytokines and GSDMD following infection by virulent EPEC.

Structural studies on the gasdermin family member GSDMA3 have uncovered a soluble pre-pore complex containing oligomers of the caspase-cleaved N-terminal fragment, which undergoes maturation upon membrane insertion (Ruan et al., 2018). Cleaved GSDMD may similarly require additional signals for membrane insertion and eventually causing cell death. Therefore, as yet unknown processes in addition to proteolytic cleavage may be required for GSDMD-mediated pyroptosis. Moreover, GSDMD pores on cell membranes can be repaired by the endosomal sorting complexes required for transport (ESCRT) machinery, including those caused by *Salmonella* infection (Rühl et al., 2018). Future studies should investigate the additional roles of caspase-1/-4 in GSDMD maturation and pyroptosis during EPEC infection in human macrophages.

On the pathogen side, inflammasome activation by EPEC was dependent on the detection of bacterial LPS and Tir-intimin-induced actin polymerization. Bacterial internalization or cytosolic escape did not correlate with macrophage survival as strains that were markedly defective in inducing pyroptosis; Δ*escF*, the strain expressing TirAA, and Δ*eae* were phagocytosed similarly to WT EPEC. Furthermore, a synthetic *E. coli* K12 strain expressing intimin, the T3SS, and Tir was fully competent in stimulating atypical pyroptosis. These findings indicated two things: (1) T3SS effectors other than Tir (and its chaperone) are not required for triggering pyroptosis and (2) activation of caspase-4 by LPS was independent of the O antigen as *E. coli* K12 expresses rough LPS.

The indispensable roles for caspase-4 and NLRP3 in detecting EPEC in MDMs adds to the diverse Gram-negative bacteria sensed by human caspase-4, including *Francisella, Legionella*, and *Yersinia* (Casson et al., 2015; Lagrange et al., 2018). These studies also found differential roles of caspase-4 in detecting natural infection by bacteria versus transfected LPS. In our study, too, atypical NLRP3 activation by natural infection of MDMs by EPEC differed from caspase-4-signaling upon LPS transfection. Unlike in *Salmonella* (Man et al., 2013) and *Yersinia*

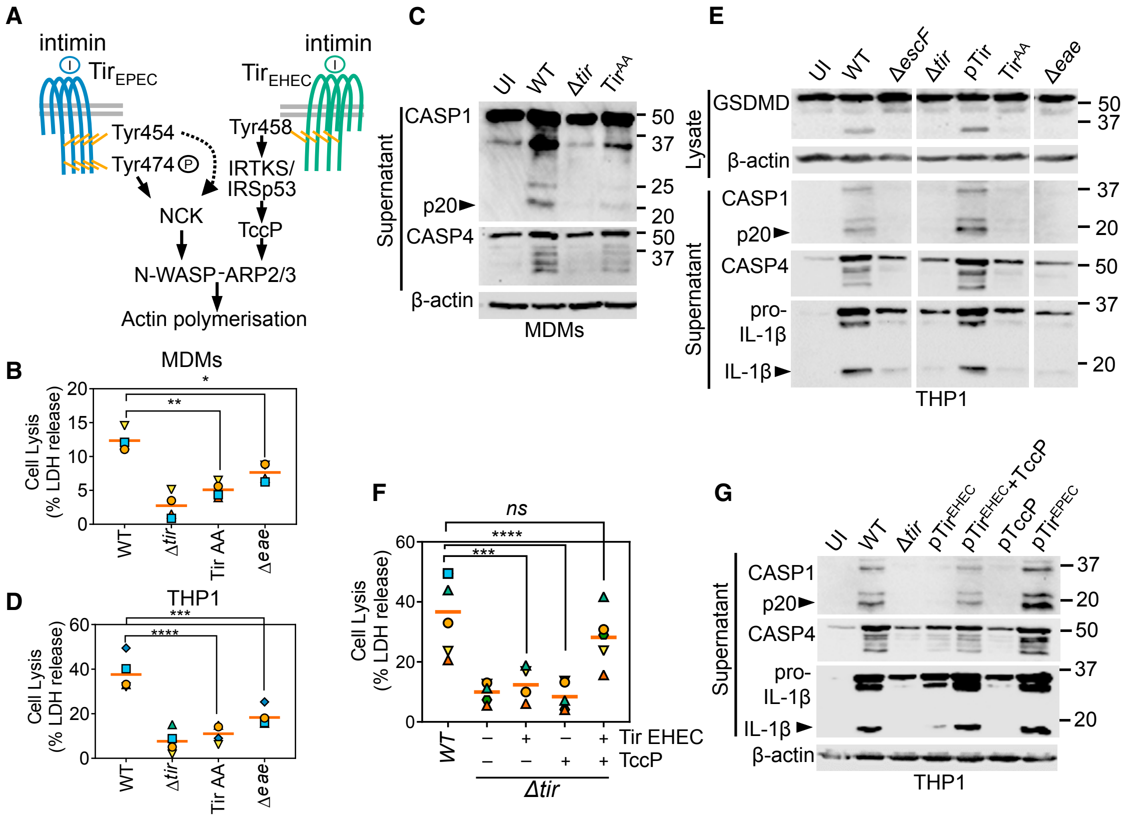

Figure 5. EPEC and EHEC Tir-Dependent Actin Polymerization Drives Pyroptosis and Cytokine Processing

(A) Schematic showing the genetically distinct mechanisms of N-WASP and ARP2/3-dependent actin polymerization induced by Tir proteins from EPEC or EHEC upon clustering by their ligand intimin.

(B and C) Cell lysis (LDH release) assays (B) and representative immunoblots (C) of primary human MDMs infected with the indicated strains of EPEC for 4 h (n = 4 independent experiments from four donors).

(D) Cell lysis of THP1 cells infected with the indicated strains of EPEC for 4 h (n = 4 independent experiments).

(E) Representative immunoblots from THP1 cells infected with the indicated strains of EPEC for 4 h. The intervening irrelevant lanes were removed, and images from the same immunoblot for each antibody are shown for groups of strains as labeled.

(F and G) Cell lysis assays (F) and representative immunoblots (G) from THP1 cells infected with the indicated strains of EPEC expressing Tir from EPEC or EHEC, or additionally expressing EHEC TccP (n = 5 independent experiments).

The matching shapes and colors of the symbols in the graphs in (B), (D), and (F) denote data from independent donors and/or experiments. Immunoblots are representative of experiments performed at least two times. *p < 0.05, **p < 0.01, ***p < 0.001, and ****p < 0.0001 by one-way ANOVA.

(Philip et al., 2014; Weng et al., 2014), caspase-8 was dispensable for EPEC-induced macrophage cell death.

Macrophage pyroptosis strictly relied on the expression of the EPEC LEE virulence regulon, which not only accelerated caspase-4-dependent inflammasome activation but also led to atypical pyroptosis. It is possible that actin polymerization by Tir-intimin signaling promotes rapid LPS internalization for caspase-4 activation. Mouse caspase-11, which can localize to sites of bacterial infection (Thurston et al., 2016), binds to actin regulatory proteins AIP1 (also called WDR1; Li et al., 2007) and Flightless I (Li et al., 2008). It is possible that EPEC-induced changes to cortical actin affect local caspase-4/-11 activity. Access of cleaved GSDMD to the plasma membrane at sites of bacterial attachment may also be limited, which may be why EPEC-induced pyroptosis required the cooperative activities of caspase-4 and caspase-1 for optimal GSDMD cleavage.

A/E pathogens typically secrete 25–30 effectors, which subvert various host processes, including opsonophagocytosis, activation of nuclear factor κB (NF-κB), mitogen-activated protein kinases (MAPKs), type I interferons, and cell death (Pearson et al., 2016; Shenoy et al., 2018). NleB, NleC, NleD, and NleE, which block NF-κB and MAPKs, may also attenuate signaling by IL-1/IL-18 cytokines produced by robust Tir-dependent inflammasome activation. We showed that NleF inhibited caspase-4 in human and mouse IECs (Pallett et al., 2017). As NLRP3 expression in IECs is low, caspase-4 contributes to pro-IL-18 conversion into mature IL-18 (Knodler et al., 2014; Pallett et al., 2017). Neutrophil influx into the colonic mucosa driven by IL-18 *in vivo* was suppressed in an NleF-dependent manner (Pallett et al., 2017). However, in macrophages, only the combined deletion of NleF and NleA/EspI (and NleH and EspO as with the ΔPP6 mutant) increased EPEC-induced pyroptosis. We reason that a higher abundance of caspase-4 and

NLRP3 in macrophages may reduce the inhibitory effects of individual EPEC effectors.

Tir-dependent actin polymerization is genetically distinct in EPEC and EHEC. Our findings suggested that Tir-proximal events were relatively less important as compared to changes in actin dynamics. Some EPEC strains can use both NCK-dependent and TccP/EspFU-dependent pathways for actin polymerization (Whale et al., 2006; Frankel and Phillips, 2008). Whether such strains trigger heightened inflammatory responses via inflammasomes should be investigated. Alterations to actin dynamics are used by the host as a pathogen-sensing mechanism; for example, *Burkholderia* TecA (Aubert et al., 2016) and *Salmonella* SopE (Keestra et al., 2013) are detected by PYRIN and NOD1, respectively. Whether caspase-4/-11 could also serve as a guardian of the host cytoskeleton during infection by Gram-negative bacteria will need further study (Mostowy and Shenoy, 2015). In summary, our study establishes an essential role for Tir-intimin-dependent actin polymerization in rapid inflammasome activation in human macrophages and has important implications for host defense against EPEC infection.

STAR★METHODS

Detailed methods are provided in the online version of this paper and include the following:

- KEY RESOURCES TABLE
- CONTACT FOR REAGENT AND RESOURCE SHARING
- EXPERIMENTAL MODEL AND SUBJECT DETAILS
  - Ethics statement
  - Preparation of primary monocyte derived macrophages (MDMs)
  - Bacterial Strains
  - Mammalian Cell Culture
- METHOD DETAILS
  - Cell Treatments and *in vitro* infection
  - Generation of bacterial strains
  - Immunoblotting
  - LDH Assays
  - PI time-course assay
  - siRNA transfections
  - Molecular cloning, protein expression and gene-silencing
  - Retroviral and Lentiviral transduction
  - Immunofluorescence analysis
  - Enzyme-linked Immunosorbent Assays
  - Antibiotic protection assays
- QUANTIFICATION AND STATISTICAL ANALYSIS
  - Statistical Analysis

SUPPLEMENTAL INFORMATION

Supplemental Information can be found online at https://doi.org/10.1016/j.celrep.2019.03.100.

ACKNOWLEDGMENTS

The authors would like to acknowledge grants from the Wellcome Trust (to G.F. and A.R.S.) and the MRC (to P.J.G., G.F., A.R.S., and the CMBI). Work in the laboratory of L.A.F. is funded by grant BIO2017-89081R from the Spanish Ministerio de Ciencia y Universidad (MICIU; AEI/FEDER, EU). G.F. and A.R.S. would like to acknowledge the MRC-funded High-Throughput Single-Cell Analysis facility at the CMBI.

AUTHOR CONTRIBUTIONS

Conceptualization, G.F. and A.R.S.; Investigation, P.J.G., J.S.-G., S.L.S., M.K., and A.R.S.; Validation, P.J.G., J.S.-G., S.L.S., M.K., and A.R.S.; Visualization, P.J.G., J.S.-G., and A.R.S.; Methodology, J.S.-G. and P.J.G.; Writing, P.J.G., G.F., and A.R.S.; Resources, O.M., D.R.-G., and L.A.F.; Supervision, G.F. and A.R.S.

DECLARATION OF INTERESTS

The authors declare no competing interests.

Received: August 13, 2018
Revised: February 20, 2019
Accepted: March 27, 2019
Published: April 23, 2019

REFERENCES

Abe, H., Tatsuno, I., Tobe, T., Okutani, A., and Sasakawa, C. (2002). Bicarbonate ion stimulates the expression of locus of enterocyte effacement-encoded genes in enterohemorrhagic Escherichia coli O157:H7. Infect. Immun. *70*, 3500–3509.

Aubert, D.F., Xu, H., Yang, J., Shi, X., Gao, W., Li, L., Bisaro, F., Chen, S., Valvano, M.A., and Shao, F. (2016). A Burkholderia Type VI Effector Deamidates Rho GTPases to Activate the Pyrin Inflammasome and Trigger Inflammation. Cell Host Microbe *19*, 664–674.

Batchelor, M., Guignot, J., Patel, A., Cummings, N., Cleary, J., Knutton, S., Holden, D.W., Connerton, I., and Frankel, G. (2004). Involvement of the intermediate filament protein cytokeratin-18 in actin pedestal formation during EPEC infection. EMBO Rep. *5*, 104–110.

Berger, C.N., Crepin, V.F., Jepson, M.A., Arbeloa, A., and Frankel, G. (2009). The mechanisms used by enteropathogenic Escherichia coli to control filopodia dynamics. Cell. Microbiol. *11*, 309–322.

Broz, P., and Dixit, V.M. (2016). Inflammasomes: mechanism of assembly, regulation and signalling. Nat. Rev. Immunol. *16*, 407–420.

Campellone, K.G., Robbins, D., and Leong, J.M. (2004). EspFU is a translocated EHEC effector that interacts with Tir and N-WASP and promotes Nck-independent actin assembly. Dev. Cell *7*, 217–228.

Casson, C.N., Yu, J., Reyes, V.M., Taschuk, F.O., Yadav, A., Copenhaver, A.M., Nguyen, H.T., Collman, R.G., and Shin, S. (2015). Human caspase-4 mediates noncanonical inflammasome activation against gram-negative bacterial pathogens. Proc. Natl. Acad. Sci. USA *112*, 6688–6693.

Coll, R.C., Robertson, A.A., Chae, J.J., Higgins, S.C., Muñoz-Planillo, R., Inserra, M.C., Vetter, I., Dungan, L.S., Monks, B.G., Stutz, A., et al. (2015). A small-molecule inhibitor of the NLRP3 inflammasome for the treatment of inflammatory diseases. Nat. Med. *21*, 248–255.

Dahan, S., Wiles, S., La Ragione, R.M., Best, A., Woodward, M.J., Stevens, M.P., Shaw, R.K., Chong, Y., Knutton, S., Phillips, A., and Frankel, G. (2005). EspJ is a prophage-carried type III effector protein of attaching and effacing pathogens that modulates infection dynamics. Infect. Immun. *73*, 679–686.

Datsenko, K.A., and Wanner, B.L. (2000). One-step inactivation of chromosomal genes in Escherichia coli K-12 using PCR products. Proc. Natl. Acad. Sci. USA *97*, 6640–6645.

DeVinney, R., Puente, J.L., Gauthier, A., Goosney, D., and Finlay, B.B. (2001). Enterohaemorrhagic and enteropathogenic Escherichia coli use a different Tir-based mechanism for pedestal formation. Mol. Microbiol. *41*, 1445–1458.

Eldridge, M.J., and Shenoy, A.R. (2015). Antimicrobial inflammasomes: unified signalling against diverse bacterial pathogens. Curr. Opin. Microbiol. *23*, 32–41.

Eldridge, M.J.G., Sanchez-Garrido, J., Hoben, G.F., Goddard, P.J., and Shenoy, A.R. (2017). The Atypical Ubiquitin E2 Conjugase UBE2L3 Is an Indirect Caspase-1 Target and Controls IL-1β Secretion by Inflammasomes. Cell Rep. *18*, 1285–1297.

Elliott, S.J., Wainwright, L.A., McDaniel, T.K., Jarvis, K.G., Deng, Y.K., Lai, L.C., McNamara, B.P., Donnenberg, M.S., and Kaper, J.B. (1998). The complete sequence of the locus of enterocyte effacement (LEE) from enteropathogenic Escherichia coli E2348/69. Mol. Microbiol. *28*, 1–4.

Fellmann, C., Hoffmann, T., Sridhar, V., Hopfgartner, B., Muhar, M., Roth, M., Lai, D.Y., Barbosa, I.A., Kwon, J.S., Guan, Y., et al. (2013). An optimized microRNA backbone for effective single-copy RNAi. Cell Rep. *5*, 1704–1713.

Frankel, G., and Phillips, A.D. (2008). Attaching effacing Escherichia coli and paradigms of Tir-triggered actin polymerization: getting off the pedestal. Cell. Microbiol. *10*, 549–556.

Frankel, G., Phillips, A.D., Rosenshine, I., Dougan, G., Kaper, J.B., and Knutton, S. (1998). Enteropathogenic and enterohaemorrhagic Escherichia coli: more subversive elements. Mol. Microbiol. *30*, 911–921.

Furniss, R.C.D., and Clements, A. (2017). Regulation of the Locus of Enterocyte Effacement in Attaching and Effacing Pathogens. J. Bacteriol. *200*, e00336-17.

Gaidt, M.M., Ebert, T.S., Chauhan, D., Schmidt, T., Schmid-Burgk, J.L., Rapino, F., Robertson, A.A., Cooper, M.A., Graf, T., and Hornung, V. (2016). Human Monocytes Engage an Alternative Inflammasome Pathway. Immunity *44*, 833–846.

Gaidt, M.M., Ebert, T.S., Chauhan, D., Ramshorn, K., Pinci, F., Zuber, S., O'Duill, F., Schmid-Burgk, J.L., Hoss, F., Buhmann, R., and et al, . (2017). The DNA Inflammasome in Human Myeloid Cells Is Initiated by a STING-Cell Death Program Upstream of NLRP3. Cell *171*, 1110–1124.e18.

Garmendia, J., Phillips, A.D., Carlier, M.F., Chong, Y., Schüller, S., Marches, O., Dahan, S., Oswald, E., Shaw, R.K., Knutton, S., and Frankel, G. (2004). TccP is an enterohaemorrhagic Escherichia coli O157:H7 type III effector protein that couples Tir to the actin-cytoskeleton. Cell. Microbiol. *6*, 1167–1183.

Gurung, P., Malireddi, R.K., Anand, P.K., Demon, D., Vande Walle, L., Liu, Z., Vogel, P., Lamkanfi, M., and Kanneganti, T.D. (2012). Toll or interleukin-1 receptor (TIR) domain-containing adaptor inducing interferon-β (TRIF)-mediated caspase-11 protease production integrates Toll-like receptor 4 (TLR4) protein- and Nlrp3 inflammasome-mediated host defense against enteropathogens. J. Biol. Chem. *287*, 34474–34483.

Kailasan Vanaja, S., Rathinam, V.A., Atianand, M.K., Kalantari, P., Skehan, B., Fitzgerald, K.A., and Leong, J.M. (2014). Bacterial RNA:DNA hybrids are activators of the NLRP3 inflammasome. Proc. Natl. Acad. Sci. USA *111*, 7765–7770.

Kayagaki, N., Wong, M.T., Stowe, I.B., Ramani, S.R., Gonzalez, L.C., Akashi-Takamura, S., Miyake, K., Zhang, J., Lee, W.P., Muszyński, A., et al. (2013). Noncanonical inflammasome activation by intracellular LPS independent of TLR4. Science *341*, 1246–1249.

Kayagaki, N., Stowe, I.B., Lee, B.L., O'Rourke, K., Anderson, K., Warming, S., Cuellar, T., Haley, B., Roose-Girma, M., Phung, Q.T., et al. (2015). Caspase-11 cleaves gasdermin D for non-canonical inflammasome signalling. Nature *526*, 666–671.

Keestra, A.M., Winter, M.G., Auburger, J.J., Frässle, S.P., Xavier, M.N., Winter, S.E., Kim, A., Poon, V., Ravesloot, M.M., Waldenmaier, J.F., et al. (2013). Manipulation of small Rho GTPases is a pathogen-induced process detected by NOD1. Nature *496*, 233–237.

Kenny, B., DeVinney, R., Stein, M., Reinscheid, D.J., Frey, E.A., and Finlay, B.B. (1997). Enteropathogenic E. coli (EPEC) transfers its receptor for intimate adherence into mammalian cells. Cell *91*, 511–520.

Klein, J.A., Powers, T.R., and Knodler, L.A. (2017). Measurement of Salmonella enterica Internalization and Vacuole Lysis in Epithelial Cells. Methods Mol. Biol. *1519*, 285–296.

Knodler, L.A., Crowley, S.M., Sham, H.P., Yang, H., Wrande, M., Ma, C., Ernst, R.K., Steele-Mortimer, O., Celli, J., and Vallance, B.A. (2014). Noncanonical inflammasome activation of caspase-4/caspase-11 mediates epithelial defenses against enteric bacterial pathogens. Cell Host Microbe *16*, 249–256.

Lagrange, B., Benaoudia, S., Wallet, P., Magnotti, F., Provost, A., Michal, F., Martin, A., Di Lorenzo, F., Py, B.F., Molinaro, A., and Henry, T. (2018). Human caspase-4 detects tetra-acylated LPS and cytosolic Francisella and functions differently from murine caspase-11. Nat. Commun. *9*, 242.

Lai, Y., Rosenshine, I., Leong, J.M., and Frankel, G. (2013). Intimate host attachment: enteropathogenic and enterohaemorrhagic Escherichia coli. Cell. Microbiol. *15*, 1796–1808.

Levine, M.M., Bergquist, E.J., Nalin, D.R., Waterman, D.H., Hornick, R.B., Young, C.R., and Sotman, S. (1978). Escherichia coli strains that cause diarrhoea but do not produce heat-labile or heat-stable enterotoxins and are non-invasive. Lancet *1*, 1119–1122.

Li, J., Brieher, W.M., Scimone, M.L., Kang, S.J., Zhu, H., Yin, H., von Andrian, U.H., Mitchison, T., and Yuan, J. (2007). Caspase-11 regulates cell migration by promoting Aip1-Cofilin-mediated actin depolymerization. Nat. Cell Biol. *9*, 276–286.

Li, J., Yin, H.L., and Yuan, J. (2008). Flightless-I regulates proinflammatory caspases by selectively modulating intracellular localization and caspase activity. J. Cell Biol. *181*, 321–333.

Liu, Z., Zaki, M.H., Vogel, P., Gurung, P., Finlay, B.B., Deng, W., Lamkanfi, M., and Kanneganti, T.D. (2012). Role of inflammasomes in host defense against Citrobacter rodentium infection. J. Biol. Chem. *287*, 16955–16964.

Man, S.M., Tourlomousis, P., Hopkins, L., Monie, T.P., Fitzgerald, K.A., and Bryant, C.E. (2013). Salmonella infection induces recruitment of Caspase-8 to the inflammasome to modulate IL-1β production. J. Immunol. *191*, 5239–5246.

Marchès, O., Covarelli, V., Dahan, S., Cougoule, C., Bhatta, P., Frankel, G., and Caron, E. (2008). EspJ of enteropathogenic and enterohaemorrhagic Escherichia coli inhibits opsono-phagocytosis. Cell. Microbiol. *10*, 1104–1115.

McDaniel, T.K., Jarvis, K.G., Donnenberg, M.S., and Kaper, J.B. (1995). A genetic locus of enterocyte effacement conserved among diverse enterobacterial pathogens. Proc. Natl. Acad. Sci. USA *92*, 1664–1668.

Mostowy, S., and Shenoy, A.R. (2015). The cytoskeleton in cell-autonomous immunity: structural determinants of host defence. Nat. Rev. Immunol. *15*, 559–573.

Nordlander, S., Pott, J., and Maloy, K.J. (2014). NLRC4 expression in intestinal epithelial cells mediates protection against an enteric pathogen. Mucosal Immunol. *7*, 775–785.

Pallett, M.A., Crepin, V.F., Serafini, N., Habibzay, M., Kotik, O., Sanchez-Garrido, J., Di Santo, J.P., Shenoy, A.R., Berger, C.N., and Frankel, G. (2017). Bacterial virulence factor inhibits caspase-4/11 activation in intestinal epithelial cells. Mucosal Immunol. *10*, 602–612.

Pearson, J.S., Giogha, C., Wong Fok Lung, T., and Hartland, E.L. (2016). The Genetics of Enteropathogenic Escherichia coli Virulence. Annu. Rev. Genet. *50*, 493–513.

Philip, N.H., Dillon, C.P., Snyder, A.G., Fitzgerald, P., Wynosky-Dolfi, M.A., Zwack, E.E., Hu, B., Fitzgerald, L., Mauldin, E.A., Copenhaver, A.M., et al. (2014). Caspase-8 mediates caspase-1 processing and innate immune defense in response to bacterial blockade of NF-κB and MAPK signaling. Proc. Natl. Acad. Sci. USA *111*, 7385–7390.

Pollard, D.J., Berger, C.N., So, E.C., Yu, L., Hadavizadeh, K., Jennings, P., Tate, E.W., Choudhary, J.S., and Frankel, G. (2018). Broad-Spectrum Regulation of Nonreceptor Tyrosine Kinases by the Bacterial ADP-Ribosyltransferase EspJ. MBio *9*, e00170-18.

Pulloor, N.K., Nair, S., McCaffrey, K., Kostic, A.D., Bist, P., Weaver, J.D., Riley, A.M., Tyagi, R., Uchil, P.D., York, J.D., et al. (2014). Human genome-wide RNAi screen identifies an essential role for inositol pyrophosphates in Type-I interferon response. PLoS Pathog. *10*, e1003981.

Rathinam, V.A., Vanaja, S.K., Waggoner, L., Sokolovska, A., Becker, C., Stuart, L.M., Leong, J.M., and Fitzgerald, K.A. (2012). TRIF licenses

caspase-11-dependent NLRP3 inflammasome activation by gram-negative bacteria. Cell *150*, 606–619.

Rosenshine, I., Ruschkowski, S., and Finlay, B.B. (1996). Expression of attaching/effacing activity by enteropathogenic Escherichia coli depends on growth phase, temperature, and protein synthesis upon contact with epithelial cells. Infect. Immun. *64*, 966–973.

Ruan, J., Xia, S., Liu, X., Lieberman, J., and Wu, H. (2018). Cryo-EM structure of the gasdermin A3 membrane pore. Nature *557*, 62–67.

Ruano-Gallego, D., Álvarez, B., and Fernández, L.A. (2015). Engineering the Controlled Assembly of Filamentous Injectisomes in E. coli K-12 for Protein Translocation into Mammalian Cells. ACS Synth. Biol. *4*, 1030–1041.

Rühl, S., Shkarina, K., Demarco, B., Heilig, R., Santos, J.C., and Broz, P. (2018). ESCRT-dependent membrane repair negatively regulates pyroptosis downstream of GSDMD activation. Science *362*, 956–960.

Sanchez-Garrido, J., Sancho-Shimizu, V., and Shenoy, A.R. (2018). Regulated proteolysis of p62/SQSTM1 enables differential control of autophagy and nutrient sensing. Sci. Signal. *11*, eaat6903.

Shenoy, A.R., Wellington, D.A., Kumar, P., Kassa, H., Booth, C.J., Cresswell, P., and MacMicking, J.D. (2012). GBP5 promotes NLRP3 inflammasome assembly and immunity in mammals. Science *336*, 481–485.

Shenoy, A.R., Furniss, R.C.D., Goddard, P.J., and Clements, A. (2018). Modulation of Host Cell Processes by T3SS Effectors. Curr. Top. Microbiol. Immunol. *416*, 73–115.

Shi, J., Zhao, Y., Wang, K., Shi, X., Wang, Y., Huang, H., Zhuang, Y., Cai, T., Wang, F., and Shao, F. (2015). Cleavage of GSDMD by inflammatory caspases determines pyroptotic cell death. Nature *526*, 660–665.

Song-Zhao, G.X., Srinivasan, N., Pott, J., Baban, D., Frankel, G., and Maloy, K.J. (2014). Nlrp3 activation in the intestinal epithelium protects against a mucosal pathogen. Mucosal Immunol. *7*, 763–774.

Thurston, T.L., Matthews, S.A., Jennings, E., Alix, E., Shao, F., Shenoy, A.R., Birrell, M.A., and Holden, D.W. (2016). Growth inhibition of cytosolic Salmonella by caspase-1 and caspase-11 precedes host cell death. Nat. Commun. *7*, 13292.

Trinh, T., Jessee, J., Bloom, F., and Hirsch, V. (1994). STBL2: an Escherichia coli strain for the stable propagation of retroviral clones and direct repeat sequences. Focus *16*, 78–80.

Vanaja, S.K., Russo, A.J., Behl, B., Banerjee, I., Yankova, M., Deshmukh, S.D., and Rathinam, V.A.K. (2016). Bacterial Outer Membrane Vesicles Mediate Cytosolic Localization of LPS and Caspase-11 Activation. Cell *165*, 1106–1119.

Vingadassalom, D., Kazlauskas, A., Skehan, B., Cheng, H.C., Magoun, L., Robbins, D., Rosen, M.K., Saksela, K., and Leong, J.M. (2009). Insulin receptor tyrosine kinase substrate links the E. coli O157:H7 actin assembly effectors Tir and EspF(U) during pedestal formation. Proc. Natl. Acad. Sci. USA *106*, 6754–6759.

Vossenkämper, A., Marchès, O., Fairclough, P.D., Warnes, G., Stagg, A.J., Lindsay, J.O., Evans, P.C., Luong, A., Croft, N.M., Naik, S., et al. (2010). Inhibition of NF-κB signaling in human dendritic cells by the enteropathogenic Escherichia coli effector protein NleE. J. Immunol. *185*, 4118–4127.

Weiss, S.M., Ladwein, M., Schmidt, D., Ehinger, J., Lommel, S., Städing, K., Beutling, U., Disanza, A., Frank, R., Jänsch, L., et al. (2009). IRSp53 links the enterohemorrhagic E. coli effectors Tir and EspFU for actin pedestal formation. Cell Host Microbe *5*, 244–258.

Weng, D., Marty-Roix, R., Ganesan, S., Proulx, M.K., Vladimer, G.I., Kaiser, W.J., Mocarski, E.S., Pouliot, K., Chan, F.K., Kelliher, M.A., et al. (2014). Caspase-8 and RIP kinases regulate bacteria-induced innate immune responses and cell death. Proc. Natl. Acad. Sci. USA *111*, 7391–7396.

Whale, A.D., Garmendia, J., Gomes, T.A., and Frankel, G. (2006). A novel category of enteropathogenic Escherichia coli simultaneously utilizes the Nck and TccP pathways to induce actin remodelling. Cell. Microbiol. *8*, 999–1008.

Wilson, R.K., Shaw, R.K., Daniell, S., Knutton, S., and Frankel, G. (2001). Role of EscF, a putative needle complex protein, in the type III protein translocation system of enteropathogenic Escherichia coli. Cell. Microbiol. *3*, 753–762.

Wong, A.R., Pearson, J.S., Bright, M.D., Munera, D., Robinson, K.S., Lee, S.F., Frankel, G., and Hartland, E.L. (2011). Enteropathogenic and enterohaemorrhagic Escherichia coli: even more subversive elements. Mol. Microbiol. *80*, 1420–1438.

Wong, A.R., Raymond, B., Collins, J.W., Crepin, V.F., and Frankel, G. (2012). The enteropathogenic E. coli effector EspH promotes actin pedestal formation and elongation via WASP-interacting protein (WIP). Cell. Microbiol. *14*, 1051–1070.

Yang, J., Zhao, Y., Shi, J., and Shao, F. (2013). Human NAIP and mouse NAIP1 recognize bacterial type III secretion needle protein for inflammasome activation. Proc. Natl. Acad. Sci. USA *110*, 14408–14413.

Yang, D., He, Y., Muñoz-Planillo, R., Liu, Q., and Núñez, G. (2015). Caspase-11 Requires the Pannexin-1 Channel and the Purinergic P2X7 Pore to Mediate Pyroptosis and Endotoxic Shock. Immunity *43*, 923–932.

Yen, H., Sugimoto, N., and Tobe, T. (2015). Enteropathogenic Escherichia coli Uses NleA to Inhibit NLRP3 Inflammasome Activation. PLoS Pathog. *11*, e1005121.

Young, J.C., Clements, A., Lang, A.E., Garnett, J.A., Munera, D., Arbeloa, A., Pearson, J., Hartland, E.L., Matthews, S.J., Mousnier, A., et al. (2014). The Escherichia coli effector EspJ blocks Src kinase activity via amidation and ADP ribosylation. Nat. Commun. *5*, 5887.

Zhao, Y., and Shao, F. (2015). The NAIP-NLRC4 inflammasome in innate immune detection of bacterial flagellin and type III secretion apparatus. Immunol. Rev. *265*, 85–102.

Zhao, Y., Yang, J., Shi, J., Gong, Y.N., Lu, Q., Xu, H., Liu, L., and Shao, F. (2011). The NLRC4 inflammasome receptors for bacterial flagellin and type III secretion apparatus. Nature *477*, 596–600.

STAR★METHODS

KEY RESOURCES TABLE

REAGENT or RESOURCE	SOURCE	IDENTIFIER
Antibodies		
Monoclonal Anti-beta-Actin-Peroxidase antibody produced in mouse	Sigma-Aldrich	Cat# A3854 RRID:AB_262011
Caspase-4 (4B9) antibody	Santa Cruz Biotechnology	Cat# sc-56056, RRID:AB_781828
Mouse Anti-Human GSDMDC1 Monoclonal Antibody, Unconjugated, Clone 64-Y	Santa Cruz Biotechnology	Cat# sc-81868, RRID:AB_2263768
Rabbit anti-human GSDMD antibody (L60)	Cell Signaling Technology	Cat# 96458S
Cleaved Caspase-1 (D7F10) Rabbit Antibody	Cell Signaling Technology	Cat# 3866S RRID:AB_2069051
anti-Caspase-1 (p20) (human) mAb (Bally-1) antibody	AdipoGen	Cat# AG-20B-0048, RRID:AB_2490257
Human IL-18 Polyclonal Antibody	MBL International	Cat# PM014, RRID:AB_592017
Goat Anti-Human Il-1 beta / il-1f2 Polyclonal antibody, Unconjugated	R and D Systems	Cat# AF-201-NA, RRID:AB_354387
anti-Asc pAb (AL177) antibody	AdipoGen	Cat# AG-25B-0006, RRID:AB_2490440
Rat Anti-Human Caspase-11 Monoclonal Antibody, PE Conjugated, Clone 17D9	Thermo Fisher Scientific	Cat# 12-9935-82, RRID:AB_1518784
Tir CT	Batchelor et al., 2004	N/A
DnaK (*E. coli*), mAb (8E2/2) antibody	Enzo Life Sciences	Cat# ADI-SPA-880, RRID:AB_10619012
Anti-α0127:H6	R. La Ragione	N/A
Anti-WASL antibody produced in rabbit	Sigma-Aldrich	Cat# HPA005750, RRID:AB_1854729
Donkey Anti-Rabbit IgG, Whole Ab ECL Antibody, HRP Conjugated	GE Healthcare	Cat# NA934, RRID:AB_772206
Sheep Anti-Mouse IgG, Whole Ab ECL Antibody, HRP Conjugated	GE Healthcare	Cat# NA931, RRID:AB_772210
Donkey anti-goat IgG-HRP Polyclonal, Hrp Conjugated antibody	Santa Cruz Biotechnology	Cat# sc-2056, RRID:AB_631730
Donkey anti-Goat IgG (H+L) Secondary Antibody, HRP	Thermo Fisher Scientific	Cat# A15999, RRID:AB_2534673
Cy3-AffiniPure Fab Fragment Donkey Anti-Rabbit IgG (H+L) antibody	Jackson ImmunoResearch Labs	Cat# 711-167-003, RRID:AB_2340606
DyLight 488 AffiniPure Donkey anti Rabbit IgG (H+L) antibody	Jackson ImmunoResearch Labs	Cat# 711-485-152, RRID:AB_2492289
Alexa Fluor 488 AffiniPure Donkey Anti-Chicken IgY (IgG) (H+L) antibody	Jackson ImmunoResearch Labs	Cat# 703-545-155, RRID:AB_2340375
Alexa Fluor 647 donkey anti-mouse antibody	Jackson ImmunoResearch Labs	Cat# 715-606-151, RRID:AB_2340866
Alexa Fluor® 594 Phalloidin antibody	Thermo Fisher Scientific	Cat# A12381, RRID:AB_2315633
Alexa Fluor® 647 Phalloidin antibody	Thermo Fisher Scientific	Cat# A22287, RRID:AB_2620155
Chemicals, Peptides, and Recombinant Proteins		
LPS-EB (LPS from *E. coli* O111:B4)	Invivogen	Cat# tlrl-3pelps
ATP	Sigma-Aldrich	Cat# A2383
Chloroquine	Sigma-Alrdich	Cat# C6628
Nigericin	Sigma-Aldrich	Cat# N7143
Cytochalasin D	Sigma-Aldrich	Cat# C8273
Isopropyl β-D-1-thiogalactopyranoside (IPTG)	Sigma-Aldrich	Cat# #I6758
Kanamycin	Sigma-Aldrich	Cat# 60615
Ampicillin	Sigma-Aldrich	Cat# A9518
Chloramphenicol	Sigma-Aldrich	Cat# C0378
Puromycin dihydrochloride from *Streptomyces alboniger*	Sigma-Aldrich	Cat# P8833

(*Continued on next page*)

Continued

REAGENT or RESOURCE	SOURCE	IDENTIFIER
Gentamicin	Sigma-Aldrich	Cat # G1272
Propidium iodide (PI)	Sigma-Aldrich	Cat # P4170
MCC950	Tocris Bioscience	Cat # 5479
Probenecid	Sigma-Aldrich	Cat# P8761-25G
Lipofectamine 2000 Transfection Reagent	Life Technologies	Cat# 11668027
DMSO	Sigma	Cat# D2438-50ML
cOmplete protease inhibitor cocktail	Roche	Cat# 04693116001
Pierce Phosphatase Inhibitor Mini Tablets	Thermo Fisher Scientific	Cat# A32957
Pierce Protease Inhibitor Mini Tablets, EDTA-free	Thermo Fisher Scientific	Cat# A32955
Clarity Western ECL Blotting substrate	Bio-Rad Laboratories	Cat# 1705061
ECL Prime Western Blotting Detection Reagent	GE-Healthcare	Cat# RPN2236
DAPI for nucleic acid staining	Sigma-Aldrich	Cat# D9542
Hoechst 33342 dye	Thermo Fisher Scientific	Cat# H1399
ProLong Gold Antifade Mountant	Thermo Fisher Scientific	Cat# P36930
Phenylmethanesulfonyl fluoride	Sigma-Aldrich	Cat# P7626
Phorbol myristate acetate (PMA)	Sigma-Aldrich	Cat# P8139
HEPES solution	Sigma-Aldrich	Cat# H0887
Trypsin-EDTA	Sigma-Aldrich	Cat# T4049
Dulbecco's minimal Eagle media Low Glucose (1000mg/L)	Sigma-Aldrich	Cat# D6046
Dulbecco's minimal Eagle media High Glucose (4500mg/L)	Sigma-Aldrich	Cat# D5796
RPMI 1640	Sigma-Aldrich	Cat# R8758
RPMI 1640 – Phenol Red Free	GIBCO	Cat# 11835030
Fetal Bovine Serum	Sigma-Aldrich	Cat# F9665
Sodium pyruvate	Sigma-Aldrich	Cat# S8636
Penicillin-Streptomycin	Sigma-Aldrich	Cat# P4333
Opti-MEM	GIBCO	Cat # 31985062
L-Glutamin Solution	Sigma	Cat # G7513
CD14-Biotin	Miltenyi Biotec	Cat# 130-190-485
Anti-Biotin Microbeads	Miltenyi Biotec	Cat# 130-190-485
LS Columns	Miltenyi Biotec	Cat# 130-042-401
LeucoSep Centrifuge Tubes	Greiner Bio-One	Cat# 227288
Critical Commercial Kits		
CytoTox 96® Non-Radioactive Cytotoxicity Assay	Promega	Cat# G1780
Human Il-1 beta/Il-1F2 DuoSet	R and D Systems	Cat# DY201
Viromer Blue	Lipocalyx	Cat# VB-01LB-00
Biological Samples		
Healthy CD14 positive monocyte-derived macrophages	NHS Blood and Transplant, Colindale London, NW9 5BG	N/A
Experimental Models: Bacterial Strains		
Stbl2 *E. coli*	Trinh et al., 1994	N/A
EPEC E2348/69	Levine et al., 1978	N/A
Δ*escF* E2348/69	Wilson et al., 2001	ICC171
Δ*tir* E2348/69	Berger et al., 2009	ICC255
Δ*eae* E2348/69	Marchès et al., 2008	ICC257
E2348/69 ΔPP6::CmFRT (*nleF – nleH2 – nleA/espI*)	This Study	N/A
E2348/69 ΔPP2::Km315 (*nleH1 – espJ –cif**)	This Study	N/A
E2348/69 ΔIE2::CmFRT (*nleE2 – nleB3 – espL*)	Vossenkämper et al., 2010	ICC1062
E2348/69 ΔIE6::Km315 (*espL– nleB1– nleE*)	Vossenkämper et al., 2010	ICC1060

(*Continued on next page*)

Continued		
REAGENT or RESOURCE	SOURCE	IDENTIFIER
E2348/69 ΔPP4::CmFRT (*nleI/nleG- nleB2 –nleC – nleD*)	Vossenkämper et al., 2010	ICC240
E2348/69 $Tir_{Y454A/Y474A}$	Wong et al., 2012	ICC311
E2348/69 Tir_{Y454A}	Wong et al., 2012	ICC309
E2348/69 Tir_{Y474A}	Wong et al., 2012	ICC310
SIEC	Ruano-Gallego et al., 2015	ICC1337
SIEC-LEE5	Ruano-Gallego et al., 2015	ICC1338
siEC ΔpLEE1-LEE5	Ruano-Gallego et al., 2015	ICC1339
Experimental Model: Cell Lines		
THP-1	John MacMicking laboratory (Shenoy et al., 2012)	N/A
HEK293E	John MacMicking laboratory (Shenoy et al., 2012)	N/A
HEK293T	Manoj Krishnan laboratory (Pulloor et al., 2014)	N/A
Plasmids		
pMX-CMV-YFP-$CTRL^{miR}$ (LacZ) 5′-TCACGACGTTGT AATACGACGT-$^{3'}$	Eldridge et al., 2017	N/A
pMX-CMV-YFP-$GSDMD^{miR}$ $^{5'}$-TACACATTCATTGAGGTGCTGG-$^{3'}$	Eldridge et al., 2017	N/A
pMX-CMV-YFP-$CASP4^{miR}$ 5′-ATATCTTGTCATGGACAGTCGT-$^{3'}$	Pallett et al., 2017	N/A
pMX-CMV-YFP-ASC^{miR} $^{5'}$-CAGCTCTTCAGTTTCACACCAG-$^{3'}$	This study	N/A
pMX-mAsc-mRFP	This study	N/A
pLX-mCas11-$CASP4^{miR}$	This study	N/A
pLX-mCas11KE-$CASP4^{miR}$	This study	Cys254Ala
pLX-mCas11CM-$CASP4^{miR}$	This study	Lys62Glu,Lys63Glu,Lys64Glu
pSA10-TccP	Garmendia et al., 2004	pICC281
pSA10-EspJ	Young et al., 2014	pICC1618
pACYC-TirEHEC	DeVinney et al., 2001	pEH86
pSA10-TirEPEC	Wong et al., 2012	pICC394
pKD46	Datsenko and Wanner, 2000	ori101, *repA* 101 (ts), *araBp-gam-bet-exo, blaM*
pKD3	Datsenko and Wanner, 2000	oriRγ, *blaM*, Cm^R cassette flanked by FRT sites
pSB315	Dahan et al., 2005	Source of *aphT* cassette (Kn^R)
Oligonucleotides		
$^{5'}$ATGCTATCACCATCTTCTGTAAATTTGGGGTG TTCATGGAATTCTTTAACcacgttgtgtctcaaaatctc$^{3'}$	This study (for generating E2348/69 ΔPP2::Km315)	orf530 FRTKan315
$^{5'}$ATGCCAATCATAAAGAACTGCTTATCATCAATT AGTAACATATTACGCAAgaattccccggatccgtcgac$^{3'}$	This study (for generating E2348/69 ΔPP2::Km315)	EspJ EPEC Kan315 rev
$^{5'}$ATGAAGCTCATTCTTGCGACGCGTAATTATTATCTGG AATATGGTTTGCGGTGTAGGCTGGAGCTGCTTCG$^{3'}$	This study (for generating E2348/69 ΔPP6::CmFRT	Orf294 EPEC FRT
$^{5'}$CATCCACATTGTAAAGATCCTTTGTTGTAAGTAAGAT CTGGTACCCTAATAATACATATGAATATCCTCCTTAG$^{3'}$	This study (for generating E2348/69 ΔPP6::CmFRT	Z6020FRTrev
Software and Algorithms		
Bio-Rad Image Lab	Bio-Rad	http://www.bio-rad.com/en-uk/product/image-lab-software?ID=KRE6P5E8Z
Zen Blue	Carl Zeiss	https://www.zeiss.com/microscopy/int/products/microscope-software/zen.html
Fiji^{TM}	NIH	https://imagej.nih.gov/ij/
GraphPad Prism 7.0	GraphPad Software	https://www.graphpad.com/scientific-software/prism/

CONTACT FOR REAGENT AND RESOURCE SHARING

Further information and requests for resources and reagents should be directed to and will be fulfilled by Avinash Shenoy (a.shenoy@imperial.ac.uk).

EXPERIMENTAL MODEL AND SUBJECT DETAILS

Ethics statement

For human MDM experiments, cells were isolated from screened blood obtained from anonymous adult male and female donors to the NHS Blood and Transplant, Colindale, London. Experiments were performed in compliance and approval from the Imperial College Healthcare Tissue Bank.

Preparation of primary monocyte derived macrophages (MDMs)

Leukocytes cones from anonymous healthy platelet donors to the NHSBT were used. CD14+ cells were enriched by MACS (Magnetic-activated Cell Sorting, Miltenyi Biotec) from buffy coats prepared using 50 mL LeucoSep tubes (Greiner Bio-One). Briefly, 10 mL blood from a single donor was mixed with 50 mL pre-warmed PBS, and 30 mL was processed per LeucoSep tube. The buffy coat layer obtained by centrifugation (1000 *x*g, 20 min at room temperature on a swing-out rotor) was separated and cells washed three times with 20 mL of pre-warmed RPMI and two times with 20 mL of MACS buffer (50 mg/ml BSA, 2 mM EDTA in PBS). CD14+ cells were enriched using biotinylated anti-CD14+ antibody and anti-biotin microbeads following the manufacture's protocol (Miltenyi Biotec). Enriched cells (~85%–95% CD14+ by flow cytometry) were cultured for 7 days in RPMI containing 1 mM sodium pyruvate, 10% heat inactivated fetal bovine serum (FBS), 100 μg/ml penicillin and 100 μg/ml streptomycin, 10 mM HEPES (pH 7.5) (complete RPMI) plus 20 ng/ml human M-CSF to allow differentiation into macrophages. Media was replenished every 72 h and antibiotics and M-CSF were withdrawn 24 h before experiments.

Bacterial Strains

Bacterial strains used in this study are listed in the Key Resources Table. All EPEC mutants are derivatives of EPEC serotype O127:H6 strain E2348/69 (Levine et al., 1978). Bacteria were routinely grown in lysogeny broth (LB) with appropriate antibiotics where necessary (kanamycin (100 μg/ml); chloramphenicol (25 μg/ml); ampicillin (100 μg/ml)) in a shaking incubator at 37°C for ~18 h. When used for infection, these are indicated as LB-grown bacteria in Text and Figure S2. Unless otherwise indicated DMEM-primed EPEC, which have elevated expression of the LEE/non-LEE virulence regulon, were prepared for macrophage infections as follows: overnight LB-grown cultures were diluted 1:50 into low-glucose (1000 mg/L) Dulbecco's minimal Eagle media (DMEM) and grown statically for 3 h at 37°C in a humidified incubator with 5% CO_2. When required, isopropyl thio-galactopyranoside (IPTG) for pSA10 plasmid-encoded effector expression was added 30 min prior to use (i.e., at 2.5 h post-inoculation into DMEM). SIEC strains are derivatives of *E. coli* K-12 (MG1655Δ*fimA-H*) (Ruano-Gallego et al., 2015), and were grown overnight in LB and diluted 1:50 into fresh LB and grown statically for 3 h at 37°C in a humidified incubator with 5% CO_2. Protein expression in SIEC strains and from pSA10-plasmids was induced with IPTG (0.1 mM) 30 min prior to infection.

Mammalian Cell Culture

THP1 cells were cultured in suspension in complete RPMI 1640 and seeded on glass coverslips in 24-well plates (density $6x10^5$cells/well) for immunofluorescence, 48-well plates ($4x10^5$cells/well) for western blotting or 96-well black-wall clear-bottom plates (density $1.5x10^5$cells/well) for cell death assays. Cells were differentiated with 100 ng/mL phorbol 12-myristate 13-acetate (PMA) and for 48 h and PMA withdrawn for 24 h before experimental treatments. HEK293 cells were maintained as sub-confluent monolayers in high-glucose (4500 mg/L) DMEM plus penicillin and streptomycin, sodium pyruvate and 10% heat-inactivated FBS. Retro- or Lenti- viral plasmid-transduced cell lines were cultured with puromycin (2 μg/ml). All cell lines were maintained at 37°C and 5% CO_2, and were tested to be mycoplasma-negative (LookOut Mycoplasma PCR Detection Kit, Sigma). Cell lines were validated by short tandem repeat (STR) profiling (Microsynth AG, Switzerland).

METHOD DETAILS

Cell Treatments and *in vitro* infection

Naive MDMs or PMA-differentiated naive THP1 were used for bacterial infections i.e., without priming with TLR agonists. Macrophages were infected with bacteria at a multiplicity of infection (MOI) of 10 (verified by viable counts), and infections synchronized by centrifuged for 10 min at 750 *x*g. Gentamicin (200 μg/ml) was added 2 h post-infection and incubation continued for 4 h or as indicated in the Figure Legends. Inhibitors were added 1 h before treatment or infection at the following concentrations: Probenecid (100 μM), MCC950 (5 μM). For canonical NLRP3 activation, cells were primed with ultrapure O111:B4 LPS (250 ng/mL) for 3 h followed by nigericin (20 μM) treatment for 45 min or ATP (5 mM) for 60 min. For non-canonical inflammasome activation, cells

were left unprimed and transfected for 4 h with LPS (5 μg/mL) using Lipofectamine 2000 (1% v/w). For immunoblot analyses, cells were washed three times with serum-free RPMI and bacterial infections or treatments were carried out in OptiMEM containing 1 mM sodium pyruvate (Eldridge et al., 2017).

Generation of bacterial strains

Strains and plasmids are listed in the Key Resources Table. Mutants are derivates of EPEC O127:H7 strain E2348/69 and were generated using the λ Red recombinase method (Datsenko and Wanner, 2000) using primers listed in the Key Resources Table and pKD3 (Cm^R) or pSB315 (Kn^R) plasmids as template.

Immunoblotting

For immunoblot analyses of proteins in supernatants, proteins were precipitated at −20°C overnight in acetone, air-dried and resuspended in 2X Laemmli loading buffer plus 5% 2ME (Eldridge et al., 2017). Cell extracts were prepared in ice-cold RIPA buffer (60 mM Tris pH 8.0, 150 mM NaCl, 1% NP-40, 0.5% Na-deoxycholate, 1 mM EDTA; all from Sigma) supplemented with complete protease inhibitor tablets, and 1 mM phenylmethylsulfonyl fluoride (PMSF), followed by the addition of 5% 2ME and Laemmli loading buffer(-Sanchez-Garrido et al., 2018). Pooled culture supernatants and cell extracts were prepared by resuspending air-dried precipitates of supernatants in cell lysates prepared from respective samples. SDS-PAGE used Tris-Glycine buffers and proteins were transferred to PVDF membranes using a TransBlot semi-dry electrophoretic transfer machine (Bio-Rad). Membranes were blocked for 2 h at room temperature in 10% fat-free milk and incubated overnight (at 4°C) with antibodies as indicated in the Key Resources Table. Immunoblots were developed with Clarity Western (Bio-Rad) ECL or ECL Prime (GE).

LDH Assays

Loss of cell membrane integrity was measured by quantifying lactate dehydrogenase (LDH) activity in culture supernatants using the CytoTox 96 nonradioactive cytotoxicity assay kit following manufacturer's protocol. Briefly, absorbance at 490 nm was measured, and expressed as percentage of that of uninfected/untreated cells treated with 1% Triton X-100 (Eldridge et al., 2017).

PI time-course assay

Cells were cultured in complete RPMI media without phenol red, and EPEC cultures were primed in DMEM media without phenol red to minimize background fluorescence. Immediately prior to infection, each well was supplemented with 5 μg/mL propidium iodide (PI), and fluorescence was measured every 10 min on a POLARStar Omega plate reader (BMG Labtech). Experimental values were calculated as relative percentage of uninfected cells treated with 0.05% Triton X-100 (Eldridge et al., 2017).

siRNA transfections

ON-Target Plus SMARTpool (Dhamacon) small interfering RNA (siRNA) were used to transiently knockdown *CASP4*, *ASC* and *CASP8* in MDMs and THP1 cells (see Table S1). THP1 cells were plated in 96-well plates at a density of 8×10^4 cells/well to achieve 70% confluency and transfected with siRNA using the Viromer Blue (VB) transfection reagent following the manufacturer's protocol for suspension cells. Cells were incubated in suspension for 6 h post-transfection, then differentiated using 100 ng/ml PMA and used for experiments at 72 h post-transfection. Medium was replaced with fresh medium without PMA 24 h before experiments. MDMs were transfected with siRNA using the Viromer Blue (VB) transfection reagent following the manufacturer's protocol and used after 72 h. Media was replaced with fresh RPMI 18 h prior to experiments.

Molecular cloning, protein expression and gene-silencing

pMX-CMV-YFP retroviral plasmid for miRNA30E-based silencing (22-mer gene-specific targets or LacZ sequence as non-targeting control) were generated as described before (Fellmann et al., 2013; Eldridge et al., 2017). The retroviral plasmid pMXsIP was used to express mouse Asc fused to mRFP (pMX-mASC-RFP) via the 5′LTR in the plasmid. Stable transductants were first selected with puromycin and THP1 uniformly expressing Asc-mRFP were isolated by fluorescence-activated cell sorting. The pLX-mCas11-$CASP4^{miR}$ plasmid was generated by modifying pLentiCRISPRv2 (kind gift from Feng Zhang, Addgene #5296; 3rd generation lentiviral plasmid). mCas11 was expressed using the EFS-NS promoter, and the CASP4miR30E sequence was cloned immediately downstream. The IRE-puromycin cassette (from pMXs-IP) was cloned downstream of the Cas11-$CASP4^{miR}$ sequence to generate the pLX-mCas11-$CASP4^{miR}$ plasmid. These plasmids were transduced into the $THP^{CASP4miR}$ line for more effective silencing of endogenous caspase-4 expression. Caspase-11^{CM} (Cys254Ala) and the $Cas11^{KE}$ (Lys62Glu,Lys63Glu,Lys64Glu) mutation was generated by overlap-PCR. Plasmid-inserts cloned using PCR or oligonucleotides were verified by sequencing (GATC Biotech). miRNA30E sequences used in this study are listed in The Key Resources Table.

Retroviral and Lentiviral transduction

pCMV-MMLV-Gag-Pol (for retroviral plasmids), Lentiviral packaging plasmid 1266 (for lentiviral plasmids) and pCMV-VSV-G (gifts from Pradeep Uchil and Walther Mothes, Yale University) were used as described before (Eldridge et al., 2017) with some modifications. Briefly, 1 μg DNA at the ratio of 5:4:1 of plasmid-of-interest:MMLV-Gag-Pol:VSG-G was transfected into HEK293T cells using Lipofectamine 2000 (2.5 μL per 1 μg DNA in 12-well plates) for 48 h; for lentiviral transductions, a ratio of 3:2:1 was used. Virus con-

taining supernatants were filtered through 0.45 μm low protein binding filters (Pall Life Sciences) and 300 μL were transferred on THP1 cells plated at 5 × 10^5 cells per well in 12-well plates. Puromycin (2 μg/ml) was added 48 h after transduction and replenished until stable pools were obtained. Cells were sorted to enrich YFP+ cells as necessary on a FACS Aria III flow sorter (BD Biosciences).

Immunofluorescence analysis

Cells plated on coverslips were infected as above, washed in PBS and fixed in PBS plus 4% paraformaldehyde for 15 min, washed thrice in PBS. Cells were permeabilized for 4 min in 0.2% Triton X-100, incubated in PBS plus 50 mM NH_4Cl as a quenching agent for 10 min, washed thrice in PBS, and blocked in PBS plus 1% bovine serum albumin (BSA) for 30 min before staining with primary antibodies in 1% BSA-PBS for 45 min. Coverslips were washed twice in PBS and incubated with secondary antibodies, Phalloidin AlexaFluor647 or Phalloidin Alexa Fluor 594 and DAPI dye in 1% BSA-PBS for 30 min, washed once in PBS and once in water before being mounted in Gold Pro-Long Anti-fade medium. Epifluorescence microscopy used a Zeiss AxioImager Z1 microscope and images were processed using Fiji^{TM} image or Zen Blue software.

Enzyme-linked Immunosorbent Assays

IL-1β in culture supernatants was measured using the human IL-1β ELISA kit (R&D Systems) by following manufacturer's protocol. Samples were measured on a FLUOstar Omega microplate reader (BMG Labtech) at an absorbance of 450 nm, and absorbance at 540 nm was subtracted for well-correction.

Antibiotic protection assays

Assays were performed similarly to those described before (Klein et al., 2017; Thurston et al., 2016). THP1^{GSDMDmiR} cells (7.5 × 10^4/well) were seeded in triplicate in 96-well plates and differentiated for 48 h, after which medium without PMA or antibiotics was used. Cells were infected with various strains of EPEC at an MOI of 10 (confirmed retrospectively by plating), by centrifuging bacteria on macrophages for 10 min at 700 x*g.* Gentamicin (100 μg/ml) was added at 2 h post-infection and some wells also received chloroquine (200 μg/ml) 3 h post-infection. After an additional 1 h (i.e., 4 h post-infection), cells were washed 3 times in RPMI, lysed in 0.5% Triton X-100 for 10 min and serially diluted in sterile PBS. Dilutions were plated in triplicate on selective LB agar and colonies were enumerated. The fraction of gentamicin+chloroquine protected bacteria as percentage of total gentamicin-protected bacteria for respective wells were plotted.

QUANTIFICATION AND STATISTICAL ANALYSIS

Statistical Analysis

No statistical methods were used to determine sample size. Experiments were not randomized and investigators were allocated without blinding during experimentation and analyses. All experiments were repeated independently at least twice or as indicated in Figure Legends. For ELISA, LDH-release, PI-uptake assays and CFU experiments two to three technical replicates were used to estimate experimental mean, and biologically independent repeat experiments (indicated by *n* in Figure Legends) were carried out. MDMs from independent donors were used and sometimes independent experiments repeated on cells from the same donor on a different day with fresh cultures of bacteria, as indicated by donor number and number of experiments in Figure Legends. Means from two or more biologically independent experiments are shown in figures and were analyzed by statistical methods (indicated by *n* in Figure Legends). For immunofluorescence assays, typically ∼100-200 host cells were counted from at least 5 randomly selected fields and % cells showing events were obtained for each experiment. Mean % from n = 3-4 biologically independent experiments were compared statistically. Log-transformed (CFU experiments), logit-transformed fractions (CFU experiments) or untransformed data were found to be normally distributed (based on D'Agostino & Pearson or Shapiro Wilk normality tests), and outliers were identified using the ROUT method based on false discovery rates (FDR; Q = 5%). One-way ANOVA or paired two-tailed Student's t test were used to compare means, and if more than three comparisons were made from the same dataset, *P value*s were corrected by the Dunnett's test or the FDR approach of Benjamini, Krieger and Yekutieli (Q = 5%) as implemented in Prism (Graph Pad Software Inc., version 8.0.2). Real-time PI uptake assays from biologically independent experiments were analyzed by repeated-measures two-way ANOVA followed by FDR-based correction (Q = 5%) for indicated multiple-comparisons as implemented in Prism. Means ± SEMs are plotted unless indicated otherwise. For dot-plots, all data points are shown as symbols matched by shape and color, and the mean is indicated by a horizontal line. Data plots and statistics used Prism 8.0.2.

Cell Reports, Volume *27*

Supplemental Information

Enteropathogenic *Escherichia coli* Stimulates Effector-Driven Rapid Caspase-4 Activation in Human Macrophages

Philippa J. Goddard, Julia Sanchez-Garrido, Sabrina L. Slater, Mohini Kalyan, David Ruano-Gallego, Olivier Marchès, Luis Ángel Fernández, Gad Frankel, and Avinash R. Shenoy

Goddard *et al* Figure S1

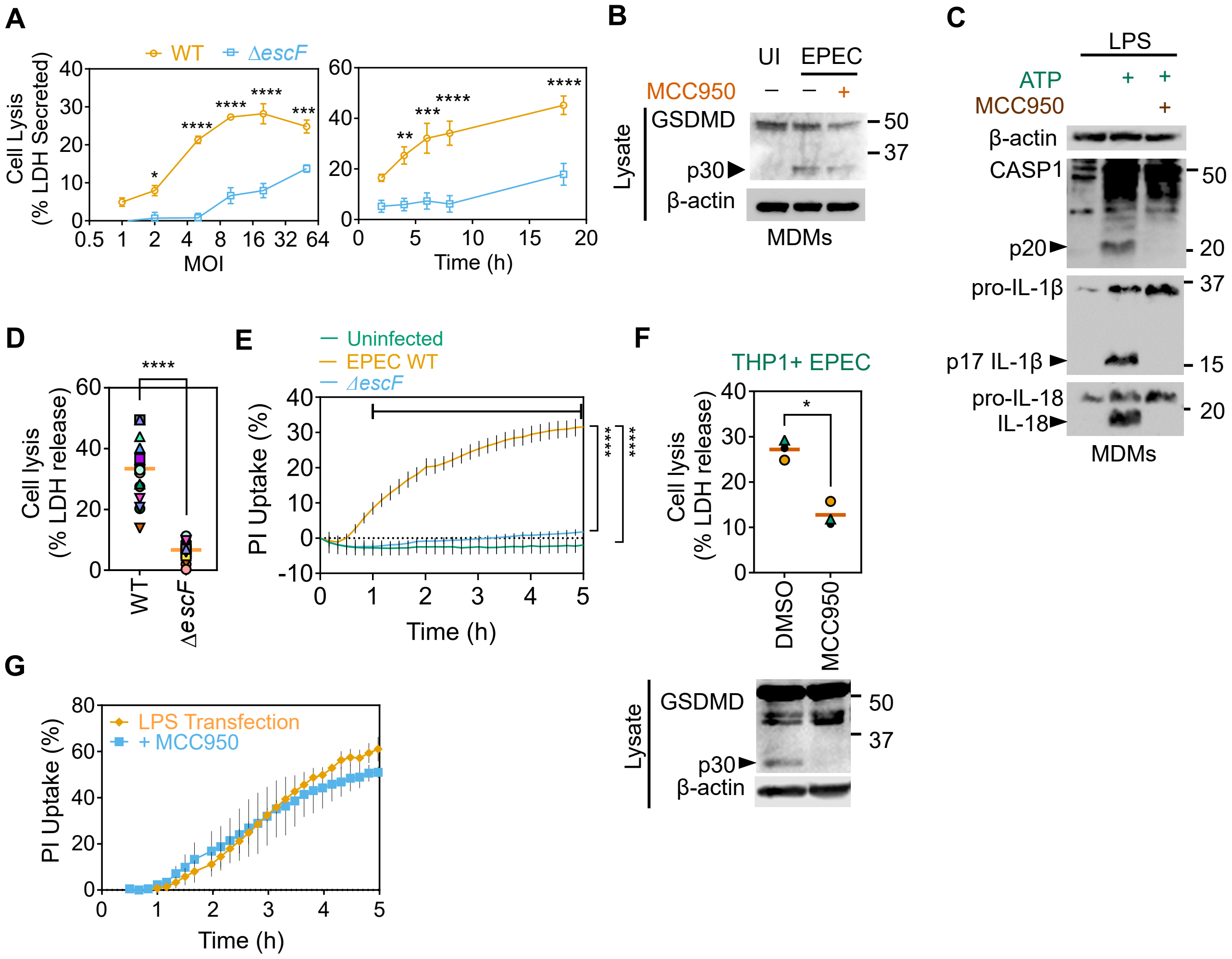

Figure S1. EPEC induces rapid NLRP3-dependent pyroptosis in THP1 cells (related to Figure 1)

(**A**) LDH-release assays from THP1 cells infected with DMEM-primed wildtype (WT) or Δ*escF* EPEC at indicated bacteria:macrophage multiplicity of infection (MOI) for 4 h (left) or at MOI 10 for indicated time (right). Mean±sem from n=3 experiments plotted. * *P<0.05,* ** *P<0.01,* *** *P<0.001,* **** *P<0.0001* by two-way ANOVA for comparisons of EPEC strains at indicated time points.

(**B**) Representative immunoblots from primary MDMs infected with EPEC in the absence (-) or presence of MCC950 (5 µM) for 4 h (*n=3* independent donors).

(**C**) Representative immunoblots from primary MDMs treated with LPS for 3 h and left untreated, or treated with ATP (5 mM; 60 min) in the absence or presence of MCC950. Western blot for β-actin was performed on cell lysates and other proteins on supernatants.

(**D-E**) Cell lysis measured as LDH release (**D**) or PI uptake (**E**) from THP1 cells left uninfected (UI) or infected with indicated wildtype (WT) or Δ*escF* EPEC strains. **** *P<0.0001* by two-tailed Student's t-test in (**D**). **** *P<0.0001* by two-way ANOVA for indicated comparisons after FDR-based correction for multiple comparisons in (**E**).

(**F**) LDH release assay (top) and representative immunoblots (below) from THP1 cells infected with EPEC in the absence (-) or presence of MCC950 (5 µM) for 4 h (*n=3* independent experiments).

(**G**) Real-time PI uptake of THP1 cells transfected with LPS using Lipofectamine 2000. MCC950 was used at 5 µM.

Matching shapes and colours of symbols in graphs in **(D)** and **(F)** denote data from independent experiments. Mean±sem plotted from *n=3* independent experiments in **(E)** and **(G)**. **P<0.05,* **** *P<0.0001* by two-tailed paired Student's *t*-test.

Goddard *et al* Figure S2

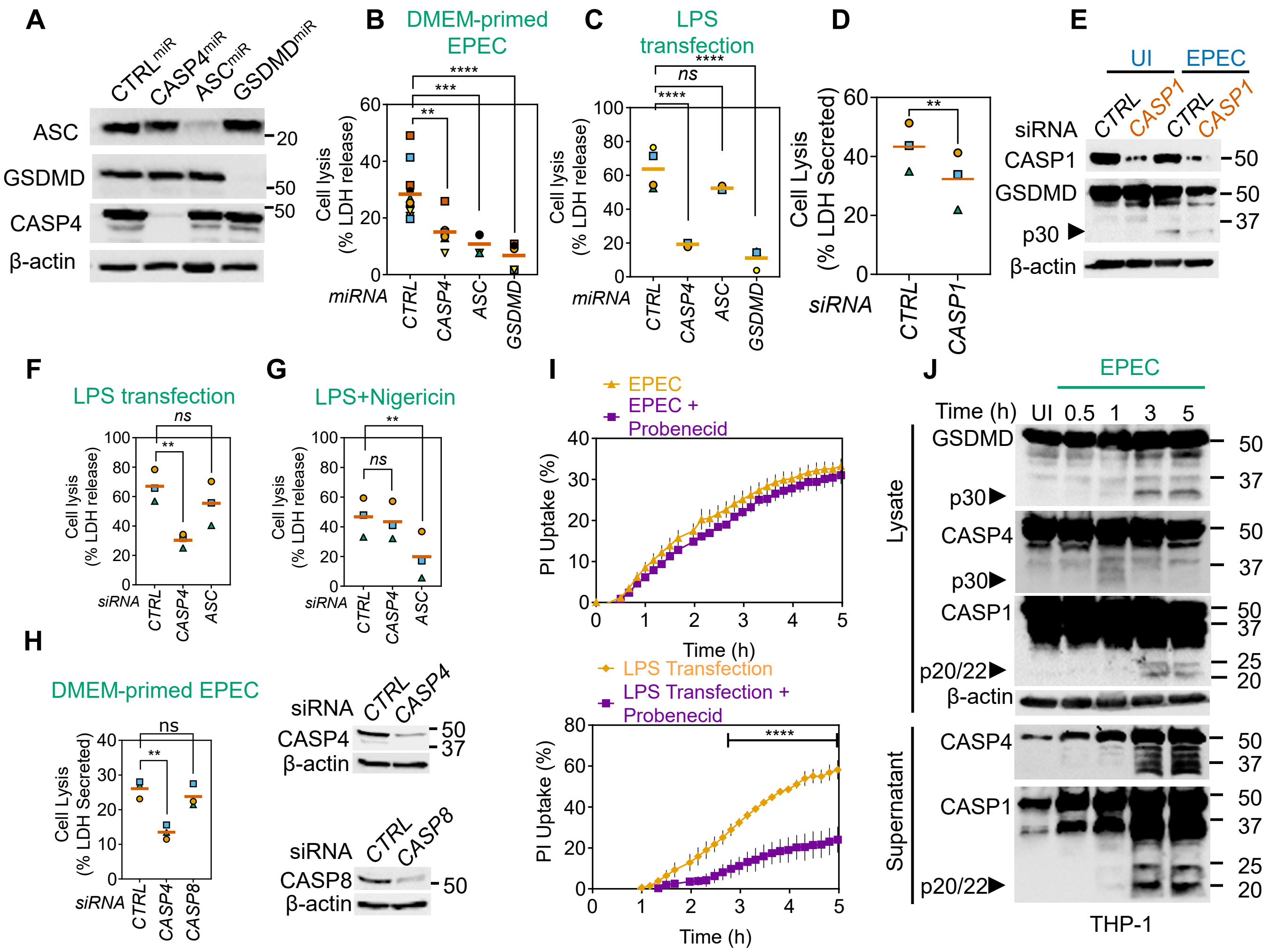

Figure S2. EPEC priming in DMEM alters caspase-4-driven pyroptosis signalling (related to Figures 2-3)
(**A**) Representative immunoblots from THP1 cells stably expressing non-targeting (CTRL) or miRNA30E (miR) against indicated genes showing silencing of protein expression.
(**B**) Cell lysis assay from indicated THP1 miRNA30E-expressing cells infected with DMEM-primed EPEC for 4 h. (*n=4-6* independent experiments)
(**C**) Cell lysis assay from indicated THP1 miRNA30E-expressing cells transfected with LPS for 4 h. (*n=3-4* independent experiments)
(**D-E**) LDH-release assay (**D**) and representative immunoblots of cell lysates (**E**) from THP1 cells transfected with non-targeting control (CTRL) or caspase-1 siRNA for 72 h and infected with EPEC (*n=3* independent experiments). ** *P<0.01* by two-tailed paired Student's *t*-test.
(**F-G**) LDH-release assay from THP1 cells transfected with indicated siRNA for 72 h and transfected with LPS in (**F**) or treated with LPS+nigericin in (**G**) (*n=3* independent experiments).
(**H**) LDH-release assay (left) and representative western blots (right) from THP1 cells transfected with indicated siRNA for 72 h followed by infection with EPEC for 4 h (*n=3* independent experiments).
(**I**) Real-time PI-uptake assay from THP1 cells either infected with DMEM-primed EPEC (top) or transfected with LPS (bottom) in the absence or presence of probenecid (100 µM). Mean±sem from *n=3* independent experiments are plotted. **** *P<0.0001* by two-way ANOVA with FDR-based correction for multiple comparisons.
(**J**) Representative immunoblots of cell lysates or supernatants of THP1 cells left uninfected (UI) or infected with DMEM-primed EPEC for indicated times.
Matching shapes and colours of symbols in graphs in (**B-D, F-H**) denote data from independent experiments. Immunoblots are representative of experiments performed at least three times. ** *P<0.01,* *** *P<0.001,* **** *P<0.0001* for indicated comparisons by one-way ANOVA in **B, C, F-H** and two-way ANOVA in **I**; ns – not significant.

Goddard *et al* Figure S3

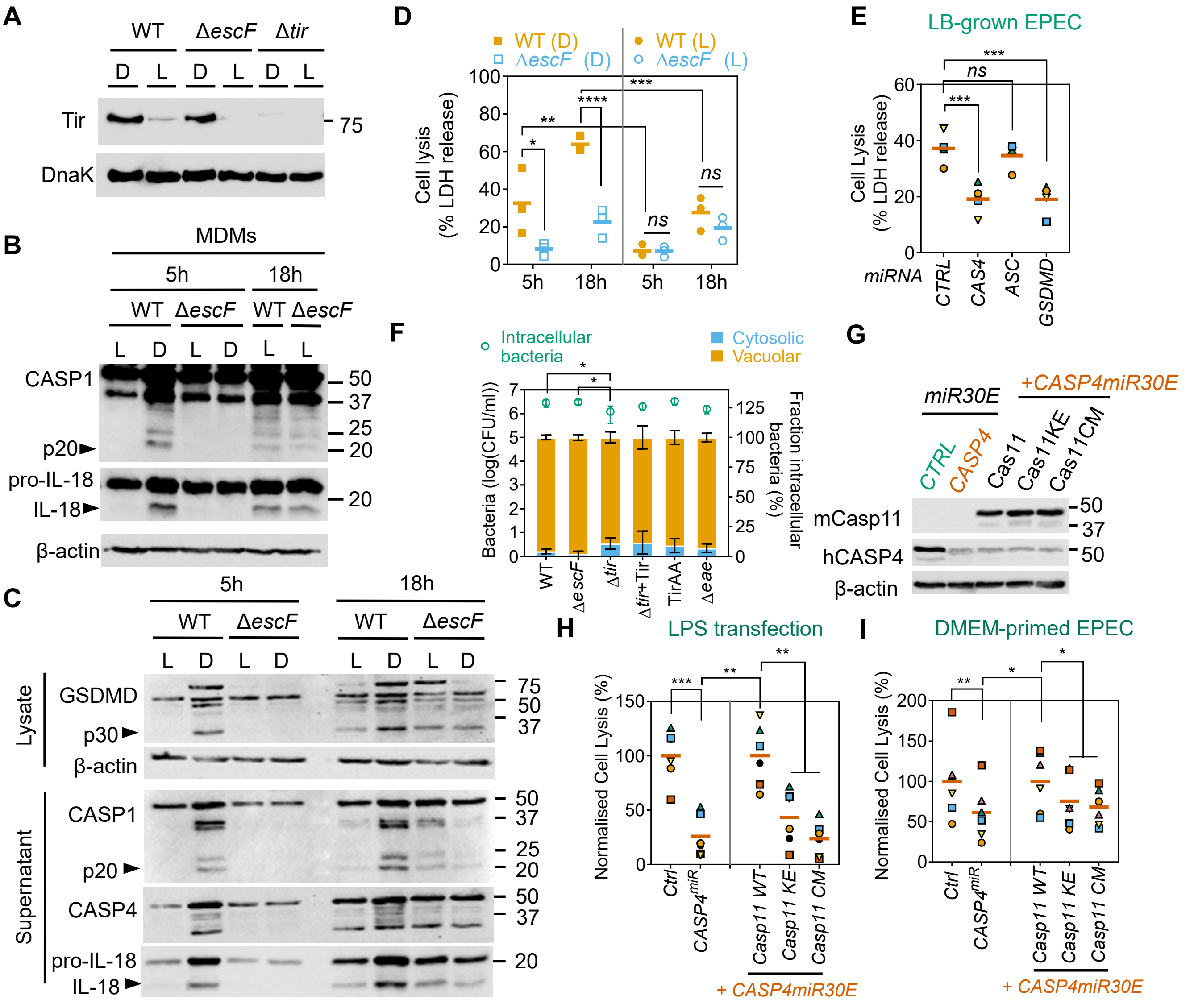

Figure S3. Virulence regulon expression influences EPEC-induced pyroptosis (related to Figures 2-3)
(**A**) Expression of Tir and DnaK (as a house-keeping gene) in indicated strains of EPEC grown with DMEM-priming (D) or in lysogeny broth (L).
(**B-C**) Representative immunoblots from MDMs (**B**) or THP1 (**C**) infected with indicated strains of EPEC grown with DMEM-priming (D) or in LB (L) at 5 h or 18 h post-infection as labelled.
(**D**) LDH release measurements from THP1 cells infected with indicated strains of EPEC (MOI 10) which were DMEM-primed (D) or LB-grown (L) after 5 h or 18 h of infection (*n=3* experiments).
(**E**) Cell lysis assay from indicated THP1 miRNA30E-expressing cells infected with LB-grown EPEC (MOI 10) for 18 h. (*n=4* independent experiments).
(**F**) Total intracellular bacteria (left axis) and fraction of intracellular bacteria (right axis) for indicated strains of EPEC at 4 h post-infection of THP1^{GSDMDmiR} cells. Mean±sem from *n*=4-6 independent experiments are plotted. Mean fractional cytosolic bacteria are similar (4-12 %) for all strains. * *P<0.05* by one-way ANOVA and Tukey's test for indicated comparisons of total intracellular bacterial CFU; means of other strains are not different when compared to WT or Δ*tir*.
(G) Representative immunoblots from THP1 cells stably expressing non-targeting (*CTRL*) or *CASP4*-miRNA30E (miR30E) and *CASP4miR* expressing cells reconstituted with indicated mCaspase-11 variants.
(H-I) Cell lysis assays from indicated THP1 cells transfected with LPS (**H**) or infected with DMEM-primed EPEC (**I**) for 4 h (*n=6* independent experiments).
Immunoblots are representative of 2-3 independent repeats. Matching shapes and colours of symbols in graphs in **E, H** and **I** denote data from independent experiments. * *P<0.05,* ** *P<0.01,* *** *P<0.001,* **** *P<0.0001* for indicated comparisons by one-way ANOVA in **E, F, H, I** and two-way ANOVA in **D**; ns – not significant.

Goddard *et al* Figure S4

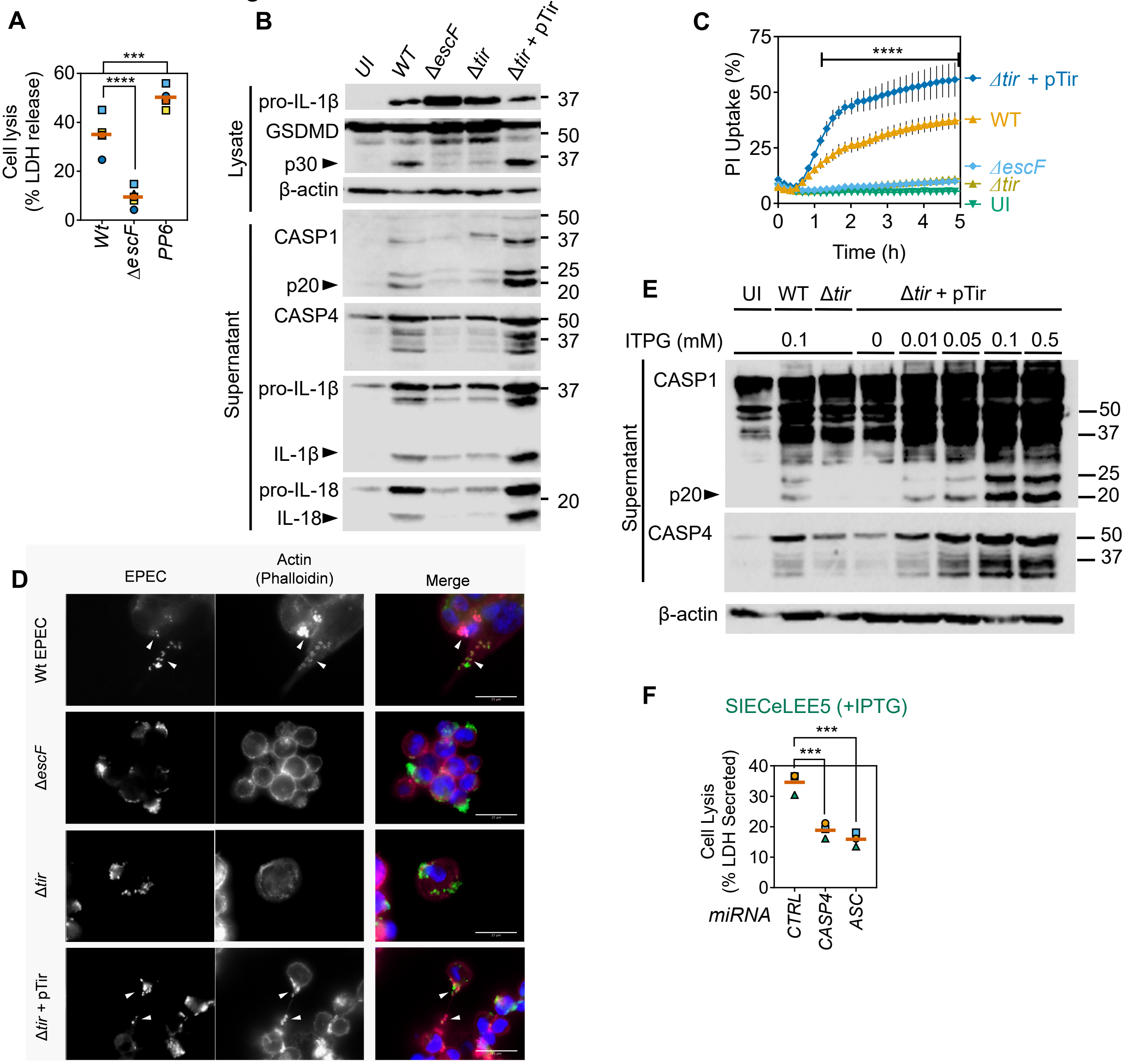

Figure S4. Tir is required for EPEC-induced inflammasome activation (related to Figure 4)

(**A**) LDH release assay from THP1 cells infected with indicated strains of EPEC for 5 h. *n=5* independent experiments. *** *P<0.001,* **** *P<0.0001* by one-way ANOVA. Matching shapes and colours of symbols in graphs denote data from independent experiments.

(**B-C**) Representative immunoblots (**B**) and PI uptake assay (**C**) from THP1 cells infected with indicated EPEC strains. IPTG (0.1 mM) was added 30 min prior to infection to induce Tir expression from pSA10 plasmid (pTir). Mean±sem from *n= 3-6* independent experiments plotted in (**C**). **** *P<0.0001* for comparisons by two-way ANOVA between WT EPEC and other strains at indicated time points after FDR-based correction for multiple comparisons.

(**D**) Representative immunofluorescence microscopy images of THP1 cells infected with indicated EPEC strains for 2 h. Arrowheads indicate actin polymerisation. Scale bar – 25 µm.

(**E**) Representative immunoblots from THP1 cells infected for 4 h with Δ*tir* EPEC expressing Tir from an IPTG-inducible plasmid (pTir). Bacteria were treated with indicated concentrations of IPTG for 30 min before infection.

(**F**) LDH-release assay from THP1 cells expressing *CTRL*, *CASP4* or *ASC* miRNA infected for 4 h with SIECeLEE5 (treated with IPTG). *** *P<0.001* by one-way ANOVA.

Matching shapes and colours of symbols in **A** and **F** denote data from independent experiments.

Immunoblots are representative of experiments performed at least two times.

Goddard *et al* Figure S5

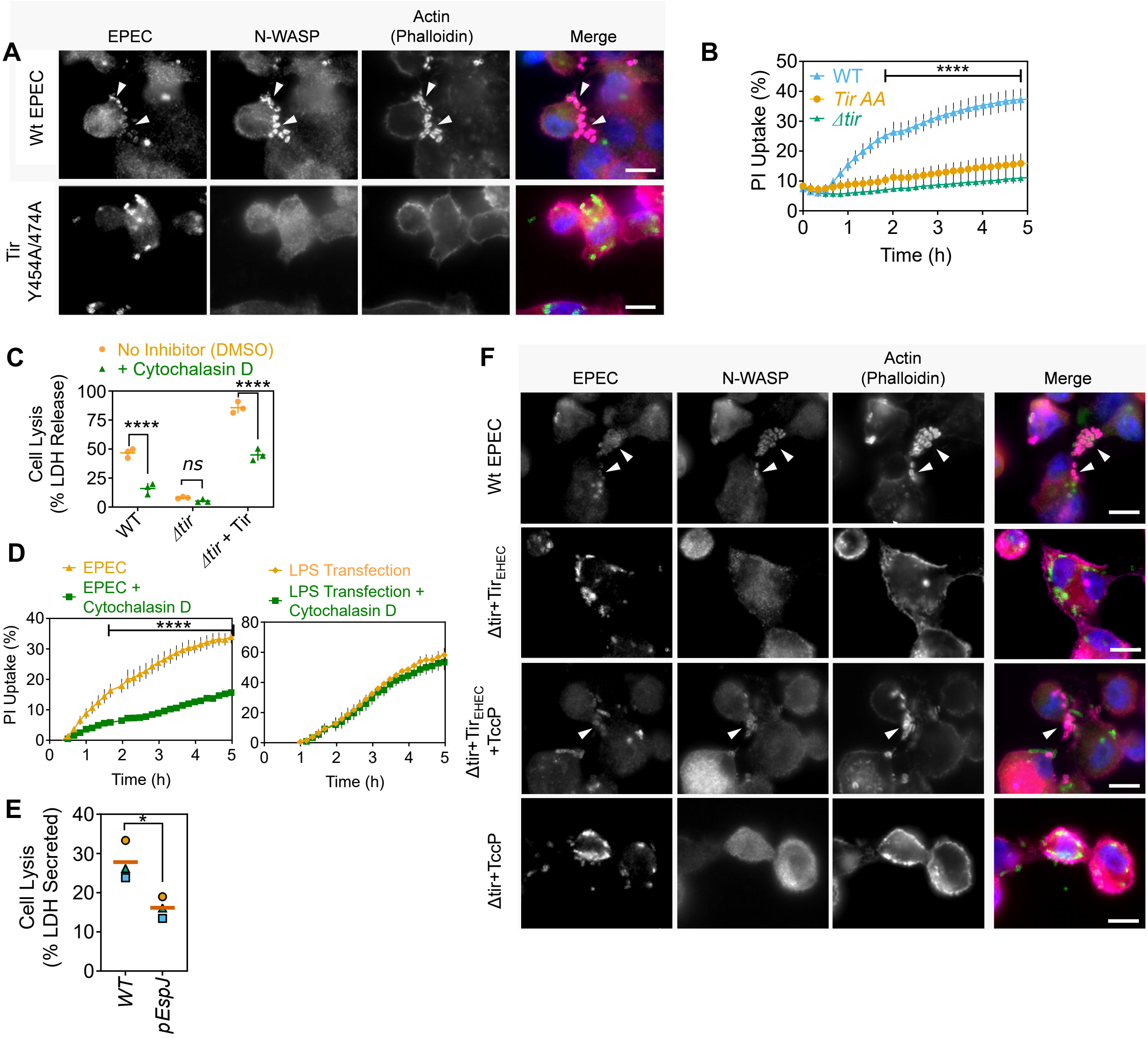

Figure S5. Tir-induced actin polymerisation drives inflammasome activation (related to Figure 5)

(**A**) Representative images from immunofluorescence microscopy of THP1 cells infected with wildtype (Wt) EPEC and EPEC expressing Tir^{AA} (Y454A/Y474A) for 2 h. Arrowheads, bacteria with actin-rich pedestals. Scale bar – 20µm.

(**B**) Real-time PI uptake assay of THP1 cells infected with indicated EPEC strains (mean±sem from *n= 3-6* independent experiments). **** *P<0.0001* by two-way ANOVA for comparisons of WT EPEC with other strains at indicated time points with FDR-based correction for multiple comparisons.

(**C**) Cell lysis measured as LDH release from THP1 cells infected with indicated EPEC strains in the absence or presence of Cytochalasin D (200 nM). *n=4* independent experiments. **** *P<0.0001, ns* – not significant by two-way ANOVA.

(**D**) Real-time PI uptake assay from THP1 cells infected with EPEC (left) or transfected with LPS (right) in the absence or presence of Cytochalasin D (200 nM). Mean±sem from *n=3* independent experiments plotted. **** *P<0.0001* for indicated comparisons by two-way ANOVA with FDR-based correction for multiple comparisons.

(**E**) Cell lysis (LDH assays) from THP1 cells infected with wildtype EPEC (WT) or a strain expressing EspJ on a plasmid (pEspJ) at MOI 10 for 4 h (*n=3* independent experiments). * *P<0.05* by two-tailed Student's *t*-test.

(**F**) Representative images from immunofluorescence microscopy of THP1 cells infected with indicated EPEC strains expressing Tir from EPEC or EHEC, or additionally expressing EHEC TccP. Arrowheads, bacteria with actin-rich pedestals. Scale bar – 20µm.

Matching shapes and colours of symbols in **E** denote data from independent experiments.

Table S1: sequences of SMART Pool siRNAs

siRNA	Source	Cat. No.
siRNA ON-TARGETplus non-targeting<br>- UGGUUUACAUGUCGACUAA -<br>- UGGUUUACAUGUUGUGUGA -<br>- UGGUUUACAUGUUUUCUGA -<br>- UGGUUUACAUGUUUUCCUA -	Dharmacon	D-001810-10<br>(D-001810-01)<br>(D-001810-02)<br>(D-001810-03)<br>(D-001810-04)
siRNA CASP4<br>- GGACUAUAGUGUAGAUGUA -<br>- CAACGUAUGGCAGGACAAA -<br>- GAACUGUGCAUGAUGAGAA -<br>- UAACAUAGACCAAAUAUCC -	Dharmacon	L-004404-00<br>(L-004404-05)<br>(L-004404-06)<br>(L-004404-07)<br>(L-004404-08)
siRNA ASC (PYCARD)<br>- GGAAGGUCCUGACGGAUGA -<br>- UCACAAACGUUGAGUGGCU -<br>- GGCCUGCACUUUAUAGACC -<br>- CCACCAACCCAAGCAAGAU -	Dharmacon	L-004378-00<br>(J-004378-06)<br>(J-004378-07)<br>(J-004378-08)<br>(J-004378-09)
siRNA CASP8<br>- GGACAAAGUUUACCAAAUG -<br>- GCCCAAACUUCACAGCAUU -<br>- GAUAAUCAACGACUAUGAA -<br>- GUCAUGCUCUAUCAGAUUU -	Dharmacon	L-003466-00<br>(J-003466-13)<br>(J-003466-14)<br>(J-003466-15)<br>(J-003466-16)
